# Exploring Extremotolerant and Extremophilic Microalgae: New Frontiers in Sustainable Biotechnological Applications

**DOI:** 10.3390/biology13090712

**Published:** 2024-09-11

**Authors:** Dorian Rojas-Villalta, David Rojas-Rodríguez, Melany Villanueva-Ilama, Rossy Guillén-Watson, Francinie Murillo-Vega, Olman Gómez-Espinoza, Kattia Núñez-Montero

**Affiliations:** 1 Biotechnology Research Center, Department of Biology, Instituto Tecnológico de Costa Rica, Cartago 159-7050, Costa Rica; rojasvillaltadorian@gmail.com (D.R.-V.); drojas21105@gmail.com (D.R.-R.); mvillanueva@estudiantec.cr (M.V.-I.); roguillen@itcr.ac.cr (R.G.-W.); frmurillo@itcr.ac.cr (F.M.-V.); 2 Facultad de Ingeniería, Universidad Autónoma de Chile, Temuco 4810101, Chile; 3 Departamento de Ciencias Químicas y Recursos Naturales, Facultad de Ingeniería y Ciencias, Universidad de La Frontera, Temuco 4811230, Chile; 4 Facultad Ciencias de la Salud, Instituto de Ciencias Aplicadas, Universidad Autónoma de Chile, Temuco 4810101, Chile

**Keywords:** extremophiles, photosynthetic microorganisms, microalgae supplementation, microalgal molecules

## Abstract

**Simple Summary:**

This review explores how certain types of microalgae, which thrive in extreme environments, can be used in various industries to promote sustainability. These microalgae, known for their ability to survive harsh conditions, produce valuable substances such as pigments, oils, and proteins. The study highlights the potential of these microorganisms to boost production efficiency, reduce contamination, and create eco-friendly products. For example, they can be used to make nutritional supplements, natural cosmetics, medicines, and biofuels. By understanding and utilizing these hardy microalgae, we can develop innovative solutions to meet the growing demand for sustainable and efficient resources, ultimately benefiting both the economy and the environment. The review concludes that investing in research on extremophilic microalgae can lead to significant advancements in biotechnology, offering new ways to address global challenges in food, energy, and healthcare.

**Abstract:**

Exploring extremotolerant and extremophilic microalgae opens new frontiers in sustainable biotechnological applications. These microorganisms thrive in extreme environments and exhibit specialized metabolic pathways, making them valuable for various industries. The study focuses on the ecological adaptation and biotechnological potential of these microalgae, highlighting their ability to produce bioactive compounds under stress conditions. The literature reveals that extremophilic microalgae can significantly enhance biomass production, reduce contamination risks in large-scale systems, and produce valuable biomolecules such as carotenoids, lipids, and proteins. These insights suggest that extremophilic microalgae have promising applications in food, pharmaceutical, cosmetic, and biofuel industries, offering sustainable and efficient alternatives to traditional resources. The review concludes that further exploration and utilization of these unique microorganisms can lead to innovative and environmentally friendly solutions in biotechnology.

## 1. Introduction

Microalgae constitute an extremely diverse group of photosynthetic microorganisms, found in nearly all ecosystems on Earth. These organisms thrive in a variety of environments, including marine water, fresh-water, desert sand, snow, ice, and hot springs [1,2,3]. Microalgae, along with cyanobacteria, are believed to be among the first photosynthetic oxygen-producing microorganisms, leading to a vast diversification in their ecology and metabolism for adaption and environment modelling [4,5]. In this context, microalgae play a key role in the health of our planet, primarily by producing a significant portion of the available oxygen [6]. Building on this ecological and evolutionary foundation, it is important to known that the ecological adaptation of microalgae has impacted in their secondary metabolism, leading to the synthesis of a diverse range of bioactive compounds [5]. Therefore, these microorganisms have been widely studied for potential biotechnological applications [7].

**Figure 1:**
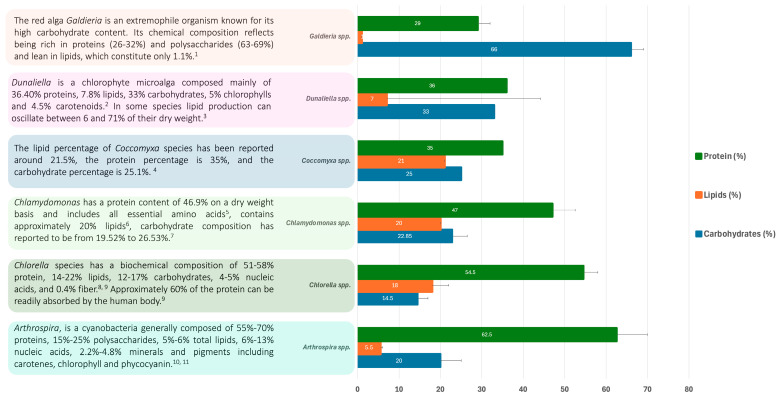
Biochemical composition of the most-studied extremotolerant and extremophilic microalgal genera based on literature data. Superscript numbers correspond to specific references cited in this document as follows: 1—Ref. [10], 2—Ref. [11], 3—Ref. [20], 4—Ref. [17], 5—Ref. [14], 6—Ref. [15], 7—Ref. [17], 8—Ref. [12], 9—Ref. [13], 10—Ref. [8], 11—Ref. [9].

The biochemical composition of microalgae varies according to the genera and species. *Arthrospira*, *Chlorella*, *Chlamydomonas*, *Coccomyxa*, *Dunaliella* and *Galdiera* are among the most widely studied genera, with reported composition percentages for the three main macromolecules (Figure 1) [8,9,10,11,12,13,14,15,16,17]. In addition, to their interesting abundance, microalgae store many secondary metabolites. These species have reported the presence of carotenoids (e.g., β-carotene, astaxanthin, canthaxanthin) in large percentage, reaching up to 14% in the *Dunaliella* genus [13,14,18,19,20,21]. Commercial utilization of carotenoids, plant growth molecules, nutritional biomass, therapeutics, pharmaceuticals, and lipids are extensively documented on microalge [22,23,24,25,26]. Several other compounds, such as bioactive lipids (e.g., with antioxidant and antimicrobial properties), phycobiliproteins (e.g., phycocyanin, phycoerythrin) and vitamins are found in microalgae [8,9,10,13,14,18,27,28,29,30]. The growing interest in microalgae has paved the way for widespread research, culminating in numerous practical applications across various industries and sectors [31]. These genera, their metabolites, and their potential biotechnological applications are explored throughout this review.

Most of the microalgae applications have been concentrated on a limited number of genera, such as *Chlorella* spp., which are adapted to mesophilic environment. However, the microalgae clade encompasses species capable of thriving under conditions that far exceed typical environmental limits (e.g., extreme pH, temperature, salinity, radiation). These organisms are considered extremophiles. In addition, such extremophilic microalgae exhibit specialized metabolic pathways as a result of selective evolutionary pressures [32]. To cope with harsh conditions, these microorganisms have developed strategies including the synthesis of bioactive compounds that hold potential for biotechnological exploitation [33]. Furthermore, the distinctive physiology of extremophilic microalgae contributes to the mitigation of contaminant risks in cost-effective, large-scale production systems, such as open photobioreactors, thereby enhancing operational viability [32,33]. Therefore, the study of these microorganisms is considered relevant for bioprospecting and industrial applications [33,34].

This review is dedicated to explaining and highlighting the promising applications of microalgae isolated from extreme environments. It aims to synthesize and elucidate the current utilizations of these microorganisms across various industries, thereby underscoring their potential as pivotal biotechnological tools. Furthermore, it amplifies research interest in this domain by demonstrating the extensive capabilities and the innovative potential of microalgae. Through a comprehensive analysis of both theoretical and practical advancements, this review aspires to foster a deeper understanding and appreciation of extremotolerant and extremophilic microalgae’s role in advancing biotechnological innovation and sustainable industrial practices.

## 2. Food Industry Applications: Extreme Microalgae Biomolecules

The increasing global population has escalated the demand of food resources for human survival. The prevalent human diet, predominantly consisting of refined grains and red meat, faces significant criticism for its adverse environmental impacts, such as pollution [35]. To mitigate this situation, it is imperative to adopt a green and sustainable food industry model. In this sense, microalgae are underexploited aquatic microorganisms of high nutritional value and represent a promising solution [36]. Microalgae, such as *Arthrospira platensis*, have been historically consumed in Asia and Africa, the Kanem people of Chad have eaten a dried algae cake made from *A. platensis* called ‘dihe’ for generations [37]. At present, this microalgae have evolved to become a promising food source with the potential to satisfy the increasing global demand [38,39]. Microalgae (e.g., *Chlorella*) can store high quantities bioactive compounds, among them proteins, lipids, polyunsaturated fatty acids, carotenoids (carotenes and xanthophylls), phycobilin, vitamins and minerals [40]. Due to their nutritional properties and their production capacity, microalgae present a significant alternative to conventional supplements and the prevailing diet [39,41]. For example, the efficiency in protein production through photobioreactors with microalgae exceeds plant cultivation or animal husbandry efficiencies [42,43].

The widespread acceptance of microalgae biomass and biomolecules has stimulated the development of novel food products from these microorganisms. Microalgae biomass has been present in the market under alternative forms. It is estimated that 75% of the annual biomass production is used exclusively in the preparation of powders, tablets, and capsules [44]. In the case of *A. platensis*, there are more than 50 kinds of *A. platensis* foods, mainly in the form of powder mixed into bread, cookies and drinks. *Arthrospira platensis* is also available in capsule and tablet form in healthy industry. Numerous *A. platensis* foods have been developed or are being developed around the world [37]. Additionally, in some countries, products based on microalgae are authorized for production, including omega-6 oils, cookies, bread, and noodles containing whole-dried microalgae, and phycoerythrin [45].

Only a limited number of microalgal strains have reached commercial success, notably including two extremophilic species: *Dunaliella* sp. and *Arthrospira* sp. (also referred to as Spirulina). The market has incorporated microalgae into a vast variety of foods in recent years. Pastas, breads, and cookies are reported to be produced using flour mixed with microalgae biomass or extracted molecules [41,46,47]. Dairy and dietary products also take advantage of the nutritional properties of microalgae, especially due to their high-protein content [48,49]. For example, incorporating Spirulina into yogurt not only accelerates the fermentation process but also enhances the yogurt’s texture, antioxidant activity, and overall nutritional profile, making it a promising natural fortification ingredient [48,49]. Interestingly, some studies have reported a consumer preference towards microalgae-fortified foods, mostly among a young demographic [50,51]. These products and other examples of mostly mesophilic microalgae incorporation in foods are extensively recent reviewed elsewhere [38,52,53].

We consider the growing market of microalgae-based products presents an opportunity to endure research regarding these microorganisms and their valuable molecules. Worldwide, between 2015 and 2019, approximately 13,090 new food products were reported to contain algae or derived components. These new products included 79% in foods and 21% in beverages [54]. In this sense, utilization of extremophilic microalgae, as exemplified by *Dunaliella* and *Arthrospira* based products (with the latest being the most widely used species), seems to gain attention in the food industry. The ability to face extreme conditions has been shown to optimize biomass production and reduce contamination by undesired species in production systems [55]. Currently, different species of extremophile microalgae, such as *A. platensis*, are ideal candidates for large-scale production due to their extreme growth conditions and their capacity to synthesize valuable and rare biomolecules under stress conditions [33,56]. These characteristics are especially relevant for open pond systems, which is a widely culture system used in the biotechnology industry prone to contamination by external microorganisms [43]. Examples of potential extremophilic microalgae and their potential application in the food industry will be explored in the following sections.

### 2.1. Protein and Amino Acid Composition

Proteins from microalgae represent an effective and sustainable food source, as these microorganisms lead in protein storage capacity and are categorized as an alternative to conventional vegetable proteins [36]. According to Khanra et al. (2018), by 2054, up to 50% of the total market will be covered by alternative proteins sources such as microalgae and insects [57]. For example, dairy and dietary products take advantage of their high protein content of microalgae. For instance, *A. platensis* is used to fortify yogurt, while microalgae like *Isochrysis galbana* and *Nannochloropsis gaditana* are incorporated into wheat bread formulations for added protein. Additionally, *Scenedesmus obliquus* is used in chocolate production, enriching it with proteins, lipids, and carbohydrates [53].

Certain microalgae have a considerably high proportion of protein in their dry biomass. Extremophilic and extremotolerant species such as *Arthrospira platensis*, able to grow in pH > 9.0 [58], and *Chlorella vulgaris* CA1, tolerant to high levels of ammonia nitrogen [59], are relevant in this regard. These species have been integrated into human nutrition due to their high-quality protein content, reaching 55–65% and >43% of the dry matter, respectively [59,60]. Other microalgae species Generally Recognized As Safe (GRAS) such as *Chlamydomonas reinhardtii* and *Euglena gracilis* present 40% of protein in their biomass [61], which makes *A. platensis* and *C. vulgaris* exceptional protein sources. Moreover, this overall percentage is a remarkably higher when compared to other plants or animals [61]. Additionally, both *Arthrospira* sp. and *Chlorella* sp. exhibit well-balanced amino acid profiles similar to other conventional protein sources, such as eggs and soybeans. According to the OMS/FAO/UNU, these microalgae comply with human essential amino acid requirements [38]. Hence, these microalgae have potential to be exploited in the food industry.

Another example is the extremophile red microalgae *Galdieria sulphuraria* CCMEE 5587.1, which can grow in acidic environments (pH 0 to 4) and above 40 °C. Under photoautotroph conditions, the microalgae present up to 44% of protein in their dry cell weight (DCW) [62]. This strain has been shown to have a high cumulative protein productivity when compared with other strain and sources such as *G. sulfuraria* strain 064/309 (~32.5%) [10], and vegetables used in the food industry, such as soy flour, parmesan cheese and skimmed milk powder (~36%) [63]. In this sense, this extremophile red microalgae biomass composition proves to be promising as a food ingredient, ideal for protein-enriched dietary applications [62].

The marine halotolerant microalga *Microchloropsis gaditana* CCMP526 also reflects industrial potential due to its high protein content and quality (40–65% in dry matter) [64,65]. In a study conducted by Qazi et al. [64], a 12% supplementation of this microalga resulted in increased levels of essential amino acids. Consequently, *M. gaditana* CCMP526 notably enhanced protein quality of produced bread. The assessment of an essential amino acid index (EAAI) revealed higher values in *Microchloropsis gaditana* (0.89–1.02, formerly *Nannochloropsis gaditana*) than egg protein (reference value) and *Arthrospira platensis* (0.81) [66]. These results reflect the potential of this marine microalga for the protein fortification of bread.

### 2.2. Lipids and Fatty Acids

Microalgae have attracted attention due to their high lipid content. Besides their primary applications in bioenergy, the promising potential of microalgal lipids in the food industry cannot be overlooked, offering a sustainable and versatile source of high-value compounds [67]. The lipid content of the overall microalgae ranges from 20% and 50% of their dry weight [68]. Specially, strains of the genus *Coccomyxa* have been researched for their lipid production capabilities [69]. Among them, the *C. melkonianii* SCCA 048 strain, resistant to heavy metal contamination [70], has captured the research community attention. A notably study revealed that by subjecting the extremophilic SCCA 048 strain to nitrogen starvation conditions increased its lipid content to approximately 40% of its dry weight [43]. Furthermore, the analysis of fatty acid methyl esters (FAME) obtained through lipid transesterification highlighted the presence of significant quantities (~85% of total lipids) of compounds that are promising for the food industry [43]. This underscores their potential utility in this sector.

Currently, the main sources of ω-3 polyunsaturated fatty acids (PUFAs) for human consumption are marine fish. However, these sources may present limitations, including low percentages of fatty acids in terms of mass, seasonal variations, and potential contamination with heavy metals [71]. In contrast, oleaginous microalgae emerge as an attractive and safe alternative, capable of naturally producing and accumulating PUFAs, including both ω-6 and ω-3 families, at significant levels [71]. PUFAs, which are hydrocarbon chains with two or more double bonds, offer great nutritional value due to their content of essential fatty acids and their high bioactive properties of interest [72]. This makes the production of PUFAs from microalgae not only relevant but promising for the formulation of new ingredients or compounds of microalgal origin in the food industry [39,73]. For example, *Schizochytrium* sp. is used in emulsion fortifications of beverages for its high content of docosahexaenoic acid (DHA), a valuable omega-3 fatty acid. Additionally, *Scenedesmus obliquus* is incorporated into chocolate, providing a rich source of lipids, along with proteins and carbohydrates [53].

The psychrophilic microalgae *Chlamydomonas malina* RCC2488 has been shown to exhibit remarkable production capacities under stress conditions, including biomass (527 mg L^−1^ day^−1^), total lipids (161.3 mg L^−1^ day^−1^) and PUFAs (85.4 mg L^−1^ day^−1^) [74]. This study underscores the extremophile strains effectiveness in biomass and metabolites production, showcasing its potential for biotechnological applications. Similarly, research on the thermophilic microalgae *Graesiella* sp. revealed that lipophilic extracts can yield a total lipid content up to 28.8% (*w*/*w*), characterized by a high abundance of fatty acids [75]. The nutritional quality of this lipids evidenced significant levels of ω-3 PUFAs (7.02%), ω-3/ω-6 ratio (0.46), hypocholesterolemic fatty acid/hypercholesterolemic fatty acid ratio index (1.39), low values of atherogenic index (0.56) and thrombogenic index (0.71) [75]. These findings highlight the potential of *Graesiella* sp. for producing compounds of high nutritional quality, suggesting its suitability for the development of natural food supplements.

### 2.3. Natural Pigments

The utilization of artificial pigments produced through chemical synthesis has raised health concerns due to the hazardous waste generated during disposal, posing risks to the environment [76]. In contrast, natural pigments derived from microalgae offer a safe alternative, exhibiting low allergenicity, toxicity and carcinogenicity, thereby minimizing health risk compared to their chemically synthesized counterparts [77]. Currently, microalgae-derived pigments, including carotenoids, chlorophylls, and phycobiliprotein, are recognized for their role as nutritional enhancers and are gaining interest as food ingredients. These pigments possess unique molecular structures and exhibit a variety of beneficial properties that differ among species [78], underscoring their potential for broader applications in food production. These beneficial properties will be explored in the following subsections.

#### 2.3.1. Carotenes and Xanthophylls

Carotenoids are lipophilic biomolecules naturally occurring with roles in colouring and photosynthesis, vary in physical, chemical, functional properties, and stability. Carotenoids are divided into primary and secondary: the primary ones actively participate in photosynthesis and are present in the photosynthetic apparatus [79] while secondary ones are usually generated in response to specific environmental stimuli and found in lipid vesicles [77]. For example, the secondary carotenoid lutein has been shown to be accumulated in the microalgae *Dunaliella salina* in response to UV-C radiation and salicylic acid, as lutein harvest blue light and favors radiation tolerance [80]. Furthermore, these biomolecules are classified according to their chemical composition into; carotenes (carotenoids composed mainly of carbon and hydrogen atoms) and xanthophylls (contain at least one oxygen atom) [81].

As humans cannot synthesize carotenoids, dietary intake is necessary [82]. Incorporating carotenoids into foods using microalgae is a promising strategy that improves the nutritional value of food and increases the product’s shelf life, due to their antioxidant capacity [33]. In this sense, the addition of *Scenedesmus almeriensis* carotenoid-rich extracts in virgin olive oils inhibited peroxidation and increased oxidative stability, improving shelf life and nutritional value [83] Commercial production of natural carotenoids has had different uses, among them, its use stands out as a food coloring (e.g., orange juice) and as additive for animal feed (poultry, fish) [31]. Apart from that, they have excellent antioxidative and preservative attributes, that help to maintain the aromas and vitamins of foods. Also, these biomolecules have a recognizable impact on the production of drinks, soups, dairy products, meats, pasta, eggs, and cakes [76,77].

##### β-Carotene

*β-Carotene*, an orange-yellow pigment, acts as a potent antioxidant and vitamin A precursor [77,84]. This carotenoid presents bioactive properties, for that reason is used throughout the world for a wide variety of purposes, including food applications [77]. For example, β-carotene is employed as an antioxidant food supplement that allows the stimulation of the immune systems against several diseases, including coronary heart disease and premature aging [85]. Some extremophiles microalgae have emerged as organisms with high potential to produce β-carotene, such as the halophilic microalga *Dunaliella salina* which accumulates up to 14% β-carotene by dry weight, outperforming synthetic variants in antioxidant activity [31]. *D. salina* β-carotene is a more powerful antioxidant than synthetic β-carotene because it contains *cis* and *trans* isomers, unlike the synthetic one that only has *trans* isomers [86]. *cis* carotenoids isomers have previously reported a higher antioxidant activity and bioaccessibility than its *trans* counterpart, resulting in a greater interest as food supplement [87]. In addition, this species has uses as a pigment for the growth environments of shrimp, trout, and ornamental fish, and due to its high bioproduction of β-carotene, it is expected to cover more than 95% of the total need for β-carotene [88]. Moreover, Tammam et al. (2011) reported that the halophilic microalga *Dunaliella tertiolecta* DCCBC26 increases β-carotene production up to ×1.4 more under hypersaline conditions, in comparison to optimal salinities [89]. Similarly, Fazeli et al. (2006) identified a greater accumulation of all-trans β-carotene at salt concentrations of 0.5 M, during exponential phase [90]. This suggested the idea of carotenoids accumulation being affected by salinity and growth stage of the microalgae [90]. These results demonstrate the potential of the high β-carotene content of *D. tertiolecta* DCCBC26 under salt stress conditions, underscoring its viability for commercialization as a natural food coloring and supplement [91].

##### Lutein

Lutein is a trending xanthophyll carotenoid with considerable attention as a valuable ingredient to the food industry, due to its strong antioxidant capabilities [79]. Extremophile microalgae are potential sources of lutein production. Indeed, *Coccomyxa acidophila* is an acidophile microalgae, that accumulates high concentrations of the antioxidant lutein [92,93]. These microalgae, particularly in mixotrophic cultures, show enhanced growth and lutein accumulation, suggesting significant biotechnological potential, especially in food and health sectors [92]. Likewise, when comparing the productivity of *C. acidophila* with other efficient lutein-producing species, such as *Scenedesmus almeriensis*, *Muriellopsis* sp. and *Chlorella protothecoides* [94], it was shown that *C. acidophila*, when grown under standard culture conditions, accumulates up to 6.1 mg·g^·1^ of dry weight, being at the upper end of the range of lutein concentrations accumulated by the mentioned microalgae [92]. This reflects the potential of this microalga in many biotechnological applications, especially in food and health-related industries [92].

In a related investigation, the impact of varying copper (Cu) concentrations on the lutein accumulation in the acidophilic microalgae *Coccomyxa onubensis* was explored. The findings revealed an enhanced growth rate and a Cu(II)-induced lutein concentration of 0.2 mM, which was 50% greater than the levels observed in control cultures [95]. Furthermore, *Mesotaenium berggrenii*, a psychrophilic green microalga, is noted for its rich carotenoid profile, including key photosynthetic pigments such as lutein and β-carotene [96]. The significant lutein content identified in these microalgae underscores their potential utility as nutritional enhancers in the food industry, suggesting their promising role in the development of naturally derived food additives. Although there are no lutein-based products from microalgae currently on the market, the existence of 108 patents reflects significant innovation, especially in genetic manipulation and extraction techniques [97].

##### Astaxanthin

Astaxanthin, another commercially valuable carotenoid derived from microalgae, exhibits potent antioxidant activity, ten times higher than that of lutein or β-carotene [98]. This molecule is highly demanded in both food and feed industries, primarily as a food-coloring agent and a natural feed additive. It is extensively used in the poultry industry aquaculture, with a significant portion of its demand stemming from salmon feed industry [31,99]. Products containing astaxanthin are already commercially available, most of them sold as food supplements containing 1–8 mg of astaxanthin [100].

The cryophilic microalga *Chlamydomonas nivalis* presents high levels of astaxanthin as one of the specialized mechanisms that give it resistance to extreme environments [101]. In this case, astaxanthin serves by reducing the light damage and photoinhibition, maximizing the photosynthetic efficiency of *C. nivalis* [101]. This microalga exhibits a notable astaxanthin content, comparable to that found in other mesophilic microalgae such as *Haematococcus pluvialis*, known for its ability to produce significant concentrations of astaxanthin under stress conditions, reaching up to 5% of the dry weight of its cells [33,102]. This suggests that microalgae species that grow naturally under stress conditions could also produce and accumulate this valuable carotenoid, being of interest for different biotechnological applications such as the food industry [33].

The green microalgae *Chlorella zofingiensis* has demonstrated a potential similar to *H. pluvialis.* This halophilic species stands out for its high mixotrophic potential, easy cultivation and ability to attain exceptionally high cell densities, positioning itself as a compelling alternative for astaxanthin production [103]. Genetic engineering is presented as a strategy to increase the content and purity of astaxanthin produced by *C. zofingiensis* [104]. Hence, obtaining greater efficiencies in purification processes allows the admission of this promising strain to the food industry [104].

##### Canthaxanthin

Similarly, canthaxanthin is a promising red/orange color carotenoid used mainly as a food dye for coloring egg yolks and chicken skin [105]. It has been reported that strains of microalgae under stress conditions produce and accumulate canthaxanthin. The *Dactylococcus dissociatus* MT1 strain isolated from an extreme environment characterized by intense solar radiation and high temperature, can produce canthaxanthin as the main secondary carotenoid under stress conditions [106]. As mentioned, canthaxanthin serves as a food pigment proven to enhance the freshness and color of animal foods, an important criterion for buyer [107]. Currently, this pigment is obtained from microbial (non-microalgae) sources, such as bacteria (e.g., *Rhodococcus maris*, *Micrococcus roseus*) and fungi (e.g., *Aspergillus carbonarius*) [108]. However, its production in microalgae has gained recent interest as it presents a higher production yield in most cases. For example, our extremophilic *D. dissociatus* MT1 reports an accumulation of 3.92 ± 1.09 mg L^−1^, a number considerably higher than the one evidenced for *Coelastrella* sp. (0.030–0.200 mg L^−1^, in heterotrophy condition), a widely study mesophilic microalgae for canthaxanthin production [106,109]. Canthaxanthin acts as a response mechanism to intense radiation, by harvesting light and increasing tolerance, hence, it is overproduced in this type of specific extremophilic microalgae such as *D. dissociatus* MT1 [106]. This shows an example of the potential of extremophilic microalgae for canthaxanthin production.

#### 2.3.2. Phycobiliproteins (PBs)

Phycobiliproteins are complexes of photosynthetic accessory pigments found in several cyanobacteria and eukaryotic algae [110]. Phycobiliproteins are widely used as food additives and notable nutritional supplements, offering various health benefits in the human diet. For example, as colorants phycobiliproteins are considered non-toxic and non-carcinogenic in counterpart to some artificial pigments as red 3 and yellow 5 [111]. Moreover, PBs present antioxidant, antimicrobial, anti-inflammatory, and immunomodulatory effects which promote its interest as food supplement [112]. Among PBs, phycocyanin stands out as one of the main ones [77]. C-phycocyanin (C-PC) is characterized by an intense blue color with potential use as a value-added food colorant. It is increasingly employed to replace synthetic pigments in a range of products, including chewing gums, candies, ice cream, and soft drinks [113,114].

Thermophilic cyanobacteria have been potentially used to produce phycobiliproteins as a potential dye in the food industry. *Synechococcus* sp. PCC 6715 strain, isolated from hot springs, is a promising source of this pigment, specifically C-phycocyanin [115]. Highly thermostable C-phycocyanins from *Synechococcus* sp. PCC 6715 offer notable advantages compared to other microalgae species such as C-phycocyanins from mesophilic *Arthrospira platensis* which, despite presenting significant advantages such as good yields and ease of cultivation, unfortunately, are limited by their low stability and rapid degradation, what affects its applicability in temperature-sensitive applications [113,116]. Given this, interest is shown in the use of thermophilic phycocyanins such as that of *Synechococcus* sp. PCC 6715 and its application as a natural pigment, which makes it a superior pigment to that of *A. platensis* [116].

Moreover, there are reports on the accumulation of phycobiliprotein present in the red microalgae *Cyanidium caldarium* and the red microalgae *Cyanidiochyzon merolae* 10D. *Cyanidium caldarium* is an acidophilic red microalga that produces the biliprotein C-phycocyanin. This microalga, when growing in acidic conditions, has the benefit of lack of competition from other photosynthetic organisms [117]. On the other hand, *Cyanidioschyzon merolae* 10D is a polyextremophilic red microalgae that produces a biomass rich in bioproducts such as thermostable phycocyanin [118]. These microalgae may be promising for the development of products in the food industry, such as pigments.

Other species belonging to the Cyanidiophyceae class such as *Galdieria phlegrea*, characterized by being a polyextremophile red alga, have been studied for their production of C-phycocyanin [119]. Carfagna et al. (2018), reported a high content of C-phycocyanin in the strains *Galdieria phlegrea* ACUF 734 (Turkey) and ACUF 009 (Italy) under autotrophic conditions [120]. The Italian strain ACUF 009 showed the highest content of C-phycocyanin, in comparison to ACUF 734 strain [120]. Likewise, the results demonstrated that by extracting heterotrophic cells from the Italian strain (ACUF 009) preheated to 70 °C for only 10 min, the thermal stability of C-phycocyanin was increased [120]. The C-phycocyanin content and stability posit *Galdieria phlegrea* ACUF 009 as a promising strain for applications in the food industry [119].

As previously mentioned, the genus *Arthrospira* comprises species that have garnered interest as promising candidates for biotechnological applications due to their nutritional potential [121]. Park et al. (2022) reported a phycocyanin purification method for the alkaliphilic cyanobacterium *Arthrospira maxima* (LIMS-PS-1691) by ultrafiltration, ion-exchange chromatography and gel filtration [122]. The process revealed a phycocyanin concentration of 1.0 mg/mL^−1^ with 97.6% purity [122]. In terms of commercial production of biomass and phycobiliproteins, *Arthrospira maxima* (LIMS-PS-1691) is considered to possess a greater potential compared to *Arthrospira platensis* [122]. This is attributed to its ability to adapt to a wider range of pH and temperature conditions.

Additionally, Adir et al. (2001) reported the crystal structure of C-phycocyanin in the thermophilic cyanobacterium *Synechococcus vulcanus* [123]. This provides information on the organization and stability of these photosynthetic proteins in extremophiles. This knowledge might help to optimize production processes, guaranteeing high quality and possible use in the food industry. Although extremophilic microalgae are not yet used for the commercial production of phycocyanin, they hold great promise. The growing global market for microalgae-based proteins and natural pigments like C-phycocyanin highlights their potential, especially given their superior stability and health benefits compared to synthetic alternatives. Extremophilic strains such as *Synechococcus* sp. and *Galdieria phlegrea* demonstrate enhanced thermostability and pigment content, making them promising candidates for future applications in the food industry.

**Figure 2:**
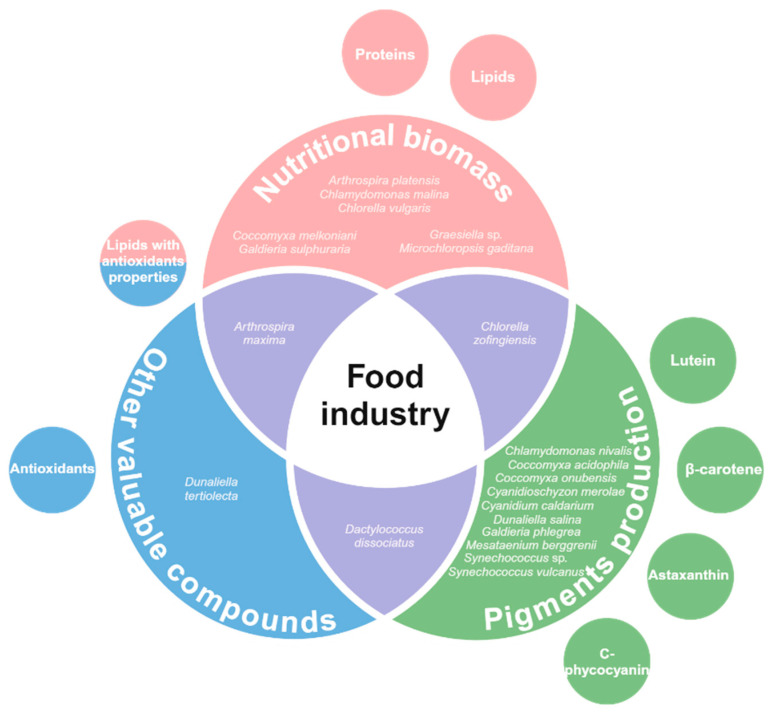
Biotechnological applications of extremophilic microalgae in the food industry. Venn diagram circles represent a summarized classification of the potential uses. Smaller circles represent examples of molecules related to corresponding application. Strains of species are ignored for simplification, please refer to Table 1 for respective strains and reference. Created with BioRender.com.

In summary, extremophilic microalgae represent a promising group for applications in the food industry (Figure 2). Their adaptability to hazardous conditions, biochemical profile, and metabolic diversity play a key role in their potential. Some species have been demonstrated to be relevant sources of molecules (e.g., proteins, lipids and antioxidants) for enhancing the nutritional potential of food products. Moreover, the production of natural pigments has also gained attention within the research community. We consider extremophilic microalgae might be a green route to compete against the negative effects of modern human diet, whilst maintaining and enhancing the nutritional aspects.

## 3. Textile and Cosmetic Industries

The cosmetic industry is one of the most developed sectors in the global market, making cosmetics essential for people’s daily use [124,125]. The influential industry significantly contributes to our economy, with retail sales reaching $60 billion in 2021. China is expected to become the leading cosmetics market by 2050, estimated approximately $450 billion [126]. Cosmetics play a crucial role in social and psychological communication, as the appearance of skin significantly influences perceptions and interactions. Studies have shown that skin care routines suing quality, effective products can enhance self-esteem and positively affect self-image [127,128]. 

Cosmetical products ensure to provide protection of skin properties and improvement of its healthy appearance [129]. Recently, due to customer concerns about chemicals in cosmetics and growing preference for environmentally friendly products has driven demand for natural-based products in this field. Extremophile microalgae, with their rich array of bioactive compounds, are emerging as promising ingredients in cosmetics [130]. These compounds, produced by microalgae to survive extreme conditions, have attracted the attention of many cosmetic companies [129]. Among these valuable bioactive compounds, carotenoids (β-carotene, astaxanthin and lutein), phycobilin, polysaccharides, fatty acids, pigments, and proteins stand out [131]. The secondary metabolites of microalgae can be used in various types of cosmetics, depending on their function. In this review we will explore sunscreens, moisturizers, anti-aging creams and colorants (face makeup) that can be obtained from extremotolerant and extremophilic microalgae. 

### 3.1. Sunscreen Products

Sunscreen products function as a barrier to human skin from ultraviolet A and ultraviolet B rays, preventing sunburn and skin cancer [131]. It is well known that UV rays cause photochemical reactions in human skin, leading to secondary effects such as the increased production of reactive oxygen species (ROS) [132]. These reactive molecules play a significant role in skin damage, contributing to cellular aging, DNA damage, and other harmful effects [133]. Most microalgae synthesize different compounds that allow them to protect themselves from the UV rays to which they and humans are exposed to. Notably, carotenoids, such as lutein are effective in shielding against UV-induced damage [129,131]. These compounds absorb and dissipate harmful UV radiation, thereby preventing it from reaching the deeper layers of the skin where it can cause long-term damage. This protective mechanism is highly beneficial in sunscreen formulations, offering a natural alternative to synthetic UV filters [134].

Carotenoids, including both xanthophylls and carotenes, have long been recognized for their antioxidant properties. Recently, lutein has attracted significant interest due to its broad range of benefits [135]. Lutein decreases reactive oxygen species production in the skin after UVR exposure [131]. Extremophilic microalgae are known to represent relevant sources of antioxidants. For example, *Chroococcidiopsis* sp. B13, a strain isolated from solar panel resistant to desiccation, radiation and UVR, presented a stable survival percentage after exposure to different doses of UV-C radiation [136]. Previously, several *Chroococcidiopsis* species have been proposed as of antioxidants producers, due to their adaptation to extreme environments [137]. This is an indicative of *Chroococcidiopsis* sp. B13 as a promising source of relevant carotenoids with antioxidant properties, although further research is required [136]. Another relevant species is *Dunaliella tertiolecta* DCCBC26, a halophilic strain mentioned in the previous section. This strain is able to produce high concentration of β-carotene, a molecule also used as an antioxidant agent [138].

High lutein content was proven in the extremophile microalgae *Coccomyxa melkonianni* SCCA 048. This is a green alga, with large production of antioxidant carotenoids and high tolerance to toxic heavy metals. This strain was isolated from the contaminated mine waters of river Ivri in the abandoned mine area of Montevecchio, SW Sardinia, Italy. This river is polluted by Cd, Co, Fe, Mn and Zn (heavy metals) with high concentration of sulphuric acid. In these conditions SCCA 048 responds by inducing the production of several antioxidants (carotenoids). Lutein is the main carotenoid produced by *C. melkonianni* SCCA 048 being 80% of total carotenoids at day 7 [139]. Similarly, the psychrophilic microalgae *Chlamydomonas nivalis* have been shown to be an alternative source of lutein [101,140]. This posits both strains as interesting for potential biotechnological applications of lutein production for cosmetic industries.

### 3.2. Moisturization

Moisturization and hydration are essential to maintain a healthy skin appearance and provide elasticity. Well-hydrated skin is better protected from skin-damaging environmental factors. As well, a proper moisturization creates a strong defense mechanism against irritants agents [141,142]. By using moisturizers on dry skin, many different disorders with symptoms of dryness are treated. Moisturizers can be considered cosmetics, but they are also supplied as drugs to treat diseases such as atopic dermatitis and ichthyosis [143]. The genus of the microalgae *Chlorella* can provide moisture and suitable viscosity. *Chorella* extracts, such as flavonoids and phenols, have antioxidant activity, moisturize, maintain skin water, stimulate collagen synthesis, and reduce wrinkle formation [131].

*Specifically*, *Chlorella vulgaris* BUACC25 was found to be rich in phenolic compounds and flavonoids. This microalga isolated from Sonapur Sea Beach, Ganjam, Odisha, can withstand high osmotic pressure and the presence of heavy metals. The UV-visible spectral peak value showed the presence of flavonoids and phenolic compounds in the crude extract. Due to its high antioxidant power, *C. vulgaris* extract showed potent antibacterial activity as well. In addition, secondary metabolites of various *Chlorella* species are mainly used as anticancer, anti-aging, anti-inflammatory, antibacterial and antifungal agents [144,145]. The amount of phenolic and flavonoids that green algae provide, can possibly be used for biotechnology applications in the manufacturing of moisturizers. 

### 3.3. Anti-Aging Creams

*Aphanothece halophytica*, a halotolerant cyanobacterium microalga known for synthesizing mycosporine-2-glycine, was isolated from Solar Lake in Sinai. In cells stressed by high nitrate levels, the production of mycosporine-2-glycine increases. This secondary metabolite, a type of mycosporine-like amino acid, inhibits the formation of advanced glycation end-products (AGEs). Consequently, it has been suggested as a potentially important compound in anti-aging strategies [146]. Saccharification of the skin, a non-enzymatic reaction between proteins, is one of the basic causes of endogenous skin aging. During the reaction, a series of complicated glycation products produced at different stages and reaction pathways are often collectively referred to as AGEs. AGEs cause the cross-linking of collagen, causing increased stiffness in the skin, which favors the appearance of wrinkles. In addition, the accumulation of brown AGEs causes hyperpigmentation [147]. Despite the significant biotechnological potential of mycosporine-2-glycine derived from *Aphanothece halophytica* in preventing AGEs, its practical application is pending further research [148,149].

### 3.4. Colorants (Makeup)

Colorants for cosmetic substances such as eye shadow, face makeup and lipstick are obtained from microalgae [141]. Pink and purple colors in cosmetics have been formulated from the natural dyes extracted from various red microalgae [150]. Proteins, such as phycoerythrin, act as a photosynthetic pigment in red algae. This protein has biotechnological applications in food science, immunodiagnostic therapy, cosmetics, protein and cell labeling, and analytical processes. B-phycoerythrin (B-PE) is an important light-harvesting phycobiliprotein in seaweeds and, due to its spectral properties, can be used as a natural dye in food, cosmetics and pharmaceuticals. Natural dyes isolated from red and blue-green algae are suitable for use in cosmetic preparations, and they are preferred as they are not considered potential toxic nor carcinogenic as their artificial counterpart.

Phycocyanin protein produced from thermophilic blue-green algae has already been formulated as eye shadow, a water-soluble phycocyanin, extracted from *Spirulina* using a phosphoric or citric acid buffer, is treated with an organic solvent like ethanol at low temperatures to create a hard soluble blue pigment. This pigment is then used in cosmetics such as eye shadow, eyeliner, or lipstick [150]. Thermophilic cyanoprokaryote *Synechococcus lividus*, isolated from thermal alkaline hot springs of Yellowstone National Park, was highlighted for the reservory of C-phycocyanin. The spectrum of monomeric C-phycocyanin is different for *Synechococcus lividus.* C-phycocyanin’s visible absorption spectrum has a maximum at 608 nm, which is blue-shifted at maximum energy higher than any others. It is not clear that this spectral change would impart any ecological advantages to the organism, but it could be of interest for use in colorants [141,151,152]. Additionally, another group of previously discussed extremophilic microalgae are relevant sources of C-phycocyanin: *Galdieria phlegrea* ACUF 009, *Cyanidium caldarium*, and *Cyanidioschyzon merolae* 10D. These microorganisms are exemplars of microalgae that carry high phycocyanin content with potential applications in the industry [117,118,153].

### 3.5. Textile Pigments

On the other side, colorants and pigments are also widely used by different productive sectors, such as the textile industry. The beginning of the textile industry goes back to a prehistoric time, where mankind began to wear clothes approximately 100,000 years ago [154]. This sector is a high income-generating industry for developing countries, such as China and Brazil [155]. It is known that the textile industry has a negative impact on the environment, therefore, there is a high necessity of going green in many production aspects [154,156]. Synthetic textile dyeing raises several human health and environmental issues. Common artificial dyes can be considered toxic and even carcinogenic for the human health [111]. Specifically, textile dyes have proven to be hard to eradicate in wastewater treatment plants, reaching open waters and bioaccumulating in fish which are later hazardous for human consumption [157]. On a commercial scale, the dyeing process consumes a gigantic amount of water and generates water with high chemical content [158]. Novel advances in biotechnology have created an interest in avoiding the use of synthetic dyes and looking for alternatives with natural resources [158]. Microalgae have emerged as promising substitutes, given the diverse and beneficial properties of their natural pigments. Notably, pigments such as carotenoids and chlorophylls derived from these organisms offer considerable potential for sustainable dyeing processes [78,158].

Chlorophyll is a green pigment fundamental to photosynthesis [57]. There are five main types of chlorophyll pigments, according to their absorption appearance: chlorophyll a, b, c, d, and f [158]. All photoautothropic organisms have chlorophyll a as part of their core reaction center of the photosynthetic system [158]. The halotolerant microalgae *Chlorella* is renowned for its high chlorophyll production, earning it the nickname ‘emerald food’ [158]. Research has demonstrated that inducing mutations in *Chlorella* can enhance its chlorophyll output. For instance, *Chlorella vulgaris* strain K, held by the Beverage Technology Research Laboratory’s culture collection, underwent mutations via ultraviolet irradiation and acriflavine treatment. These mutations targeted the mitochondrial DNA, significantly boosting chlorophyll production. Consequently, this process yielded a high-chlorophyll strain, designated *C. vulgaris* M-207A7 [144,145].

As mentioned above, β-carotene is a yellow terpenoid pigment of increasing demand and a wide variety of applications within the market [159,160]. Among the prominent microalgae producers of β-carotenoids, we remarked the green algae *Dunaliella salina* [160]. This species is notable for its extreme halotolerance, thriving in marine environments and inland salt lakes, making it the most halotolerant eukaryote identified to date [161]. In closed tubular photobioreactors, *Dunaliella salina* can achieve carotenoid concentrations up to 10% of its dry weight [160]. Under the same thread, the species *Mesataenium berggrenii* is a psychrophilic microalga recognized as a fount of β-carotene that also could exploit its use in the textile industry [96]. The utilization of microalgal carotenoids as textile dyes has expanded the biotechnology potential of both strains beyond the food industry [158]. This underscores the relevance of extremophilic microorganisms research and exemplifies their promising applications among different industries.

**Figure 3:**
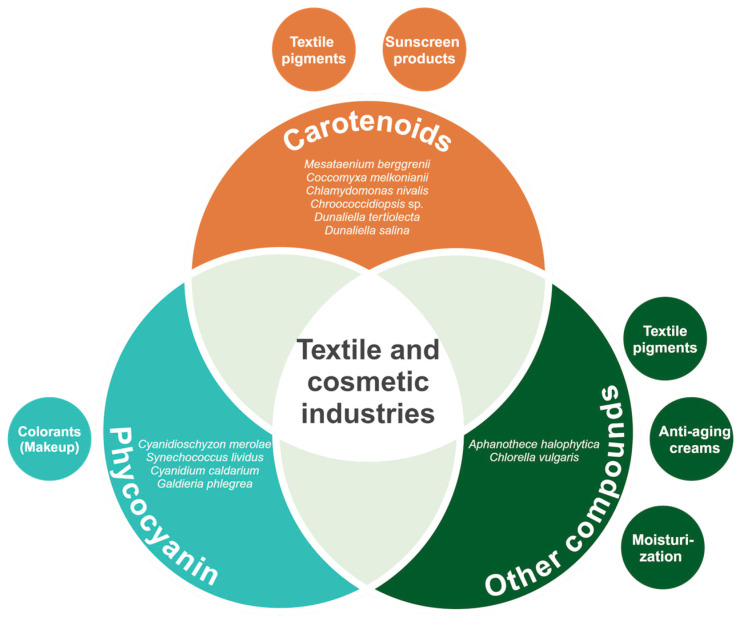
Biotechnological applications of extremophilic microalgae in the textiles and cosmetic industries. Venn diagram circles represent a summarized classification of the relevant produced molecules. Smaller circles represent examples of the application related to corresponding molecules. Strains of species are ignored for simplification, please refer to Table 1 for respective strains and reference. Created with BioRender.com.

Briefly, extremophilic microalgae and their valuable molecules hold great relevance for the textile and cosmetic industries (Figure 3). The production of carotenoids, phycocyanin and other compounds, such as amino acids and lipids, represents a key feature in microalgae metabolic diversity. In addition, the high abundance of the mentioned molecules within the microalgal biomass provides great potential for cost-effective extraction. The vast variety of applications of these molecules as textile pigments, colorants for makeup, and active agents of other products posit extremophilic microalgae as a hot spot for research.

## 4. Usage for Bioremediation

Industrial development and urbanization have increased waste production, strongly impacting the environment [162]. The use of conventional physicochemical techniques to remediate these contaminated environments has been losing interest due to the demand for high operating costs and secondary pollution to the atmosphere [163]. Consequently, this has required the use of more sustainable techniques, such as bioremediation, an environmental-friendly and cost-efficiency strategy [164]. In this sense, the use of microalgae in bioremediation can incur into a green economy by using the product (microalgae) for production of valuable products such as biofuels (a topic explored in the next section) [165]. Also, by using wastewater for the microalgae culture it is thought that the freshwater requirement can be reduced up to 90% [166]. This might exemplify how microalgae-based bioremediation is posit as an environmental-friendly and cost-efficient method.

Particularly, bioremediation relies on the ability of living organisms to immobilize or modify the chemical structure of xenobiotics, present in soils, sediments, water, and air, degrading them partially [163]. Extremophile microalgae are particularly notable in this field due to their robust biological features. These include high photosynthetic efficiency and adaptability to harsh environments rich in pollutants, such as heavy metals, high salinity, nutritional stress, and extreme temperatures [167]. In the field of bioremediation, these microalgae are effectively used to treat wastewater, classified into municipal, industrial and agricultural waters, providing a sustainable solution to address the contaminants present in these effluents [168]. A recent report highlighted microalgae have the advantage of growing fast and to degrade and detoxify a wide spectrum of organic and inorganic pollutants (explored throughout the section) through bio-adsorption, bioaccumulation or biodegradation [169,170].

Overall, bio-adsorption is a physicochemical process that involves the passive binding of contaminants to the cell surface of microalgae thanks to the unique and complex structure of their cell walls, which are rich in polysaccharides, proteins and lipids, which contain functional groups such as amino, hydroxyl, carboxyl, and sulfate [171]. Besides, bioaccumulation is an active process in which the contaminants accumulate inside the cells, due their intracellular structures (cell vacuoles, phytochelatins, ligands, metallothioneins) [171,172]. On the other hand, biodegradation could be an extracellular or intracellular process that breaks down contaminants with the help of enzymes to convert them into simpler and non-toxic forms [173,174]. For example, the microalgae *Scenedesmus obliquus* has proved to biodegrade up to 90% of high-toxic compounds, such as pentachlorophenol, while being able to growth in the resulting low-toxic compounds (monochlorophenol) [175].

Additionally, microalgae species are used as bioindicators of contamination and water quality. This utilization is due to three major physiological properties: (i) high sensitivity to environmental changes, (ii) easy sampling methods, and iii) well-known cosmopolitan species ecology [176]. Extremophilic microalgae, capable of surviving under hazardous conditions and several types of pollution, hold potential applications in this regard [177]. *Pinnularia aljustrelica*, a microalga isolated from acidic waters, is reported to dominate the microbial communities in samples of water with low pH and high metallic load [178]. This led to believe this microalga might serve as a bioindicator of waters polluted by acid mine drainage [178]. Moreover, another acidophilic species of this genus, *Pinnularia* braunii showed a similar pattern in water polluted by poor agricultural practices and urbanization [179]. An increasement of pollution in near streams samples enhanced the abundance of *P. braunii*, this posits the microalga as a promising bioindicator of chances in water quality [179].

The following subsections underline examples of extremophilic and extremotolerant microalgae and its ability to bioremediate several types of compounds.

### 4.1. Bioremediation of Organic Pollutants

Effluents enter aquatic ecosystems as organic pollutants that can contain different types of contaminants such as petroleum hydrocarbons, polyaromatic hydrocarbons and pesticides, which pose a threat to the terrestrial and aquatic ecosystem [168]. Several pesticides (diazinon and chlorpyrifos) have been demonstrated to possess a potential risk to aquatic organisms in freshwater bodies [180]. These are thought to negatively affect brain and liver enzyme activity of fish species, such as salmon [180]. As a method of bioremediation, microalgal species such as *Chlorella*, *Scenedesmus*, *Phormidium*, *Botryococcus*, *Chlamydomonas*, *Arthrospira*, *Oscillatoria*, *Desmodesmus*, *Nodularia*, and *Cyanothece* are employed to break down these organic pollutants [173]. The effectiveness of this process is influenced by several factors, including the size, strain, density, morphology, and biological activity of the microalgal cells, which play crucial roles in the bio-adsorption and elimination of contaminating organic compounds [181].

#### 4.1.1. Petroleum Hydrocarbons (PHs) and Polycyclic Aromatic Hydrocarbons (PAHs)

Polluting hydrocarbons, such as petroleum and its derivatives, polycyclic aromatic hydrocarbons (PAHs) and halogenated hydrocarbon compounds, are released into the environment through oil spills, wastewater and various activities, representing an important source of pollution [163]. It is well-known, these hydrocarbons are present in various environments and can impact the health of numerous organisms, as well as alter the balance of ecosystems. Among the most polluting hydrocarbons to the environment is the oil refinery, emitting toxic chemicals (PHs and PAHs) that affect different trophic levels [182,183].

Certain microalgae species that inhabit extreme environments have been investigated for their potential applications in the bioremediation of contaminating hydrocarbons. For example, *Nannochloropsis oculate* is a marine microalga that has the ability to grow in hypersaline water. A study carried out by Marques et al. (2021) evaluated the removal potential of PAHs and organic compounds for the treatment of water produced by oil, showing a efficiency of 94% removal of PAHs and 89-99% of organic compounds such as naphthalene, benzo(a)pyrene, benzo(b)fluoranthene, and acenaphthylene, demonstrating that this strain has the potential to remove PAHs for petroleum produced water treatment [184].

Moreover, the acidophilic microalgae *Cyanidium caldarium*, showed the capacity of growing in soils contaminated with PAHs [185]. Díaz et al. (2015) measured the chlorophyll a concentration to determine an increasement in the biomass of the microalga. The results indicated the growth of this specie, reflecting its potential as a bioindicator and its potential use in bioremediation [185].

#### 4.1.2. Pesticides

Agricultural activities are positioned as one of the main sources of pollution in surface and groundwater due to the increase in the use of synthetic pesticides (e.g., organophosphates, carbamates). This problem is exacerbated by run-off and drainage, which contribute significantly to the introduction of these polluting compounds into water bodies [186]. Although pesticides are crucial for agricultural productivity, they have been identified as potential endocrine-disrupting compounds. This might result in reproductivity and health alterations in organisms, such as affected steroid-hormone-synthetizing enzymes, immature sperm, among others [187]. Their widespread use is concerning because they can enter the food chain and bioaccumulate in organisms at higher trophic levels. Moreover, it is estimated that approximately 95% of applied pesticides do not reach their target pests but are instead deposited into the surrounding environment [170,188]. This issue is particularly acute in major agricultural nations such as China, which was the world’s leading consumer of pesticides, using 1.46 million tons in 2005 alone [189]. In response, technologies like microalgae-based treatment systems have been developed to effectively remediate pesticide contamination [186]. Microalgae pesticide removal depends on optimal microbiome conditions and biological activity, the chemical structure of the pesticide and factors related to microalgae [170].

Recently, in a study performed by Nicodemus et al. (2020) the psychrotolerant microalga *Cocomyxa subellipsoidea* C-169, native to polar regions, was used to degrade a mixture of organophosphate pesticides (diazinon, malathion and paraoxon) [190]. As a result, an efficient degradation of the pesticide mixture was obtained, where the microalgae could convert them into their intermediates (paraoxon → p-nitrophenol) through the hydrolysis pathway. Furthermore, the study demonstrates that organophosphate levels during the experimental period of 8 to 10 days decreased to less than 0.1 mg/mL and were hydrolyzed by a nucleophilic reaction dependent on reactive oxygen species (ROS) with no toxic effects on the microalgae. This exemplifies how extremophilic microalgae might be a relevant option for bioremediation processes of such hazardous compounds.

### 4.2. Bioremediation of Inorganic Pollutants

Inorganic compounds include minerals, gases, heavy metals, radioactive substances, etc., which have increased due to human activities such as mining, fuel production, energy production, household, farming, and industrial wastes [173]. Industrial development has significantly increased the release of toxic waste into the environment, mainly into soil and water [191]. Here we explore examples of extremophilic and extremotolerant microalgae able to eradicate these compounds.

#### 4.2.1. Heavy Metals

The accumulation of heavy metals in the ecosystems is one of the most hazardous environmental issues, as these metals cannot be degraded into non-toxic forms. Instead, they persist in the environment and bioaccumulate in the human food chain, posing severe risks to both environmental and human health [192]. For example, heavy metals can induce alterations in the reproductive systems of both males and females by disrupting gametogenesis and increased oxidative stress, which affects fertility [193]. According to this situation, the use of microalgae represents a potential solution to counteract heavy metal contamination due to its advantages of having a rapid growth rate, being robust, as well [167]. Likewise, the ability of a microalga to remove metals is affected by factors such as metal and biomass concentrations, pH, and contact time [172].

Field tests have demonstrated that manufactured pools and meanders utilizing microalgae can remove up to 99% of dissolved and particulate metals [173]. Notably, wastewater treatment plants contribute approximately 15% of the total 6.4 tons of cadmium released into aquatic environments annually [194,195]. Cadmium, a heavy metal, is a significant environmental pollutant known for its high toxicity even at low concentrations. In response to this issue, the extremophilic green microalgae *Chlamydomonas acidophila* RT46 shows considerable potential. Its remarkable tolerance and detoxification abilities allow it to efficiently sequester cadmium within its vacuoles, highlighting its potential in the phytoremediation of water resources contaminated by heavy metals [196].

Similarly, *Coccomyxa melkonianii* SCCA 048 is an extremophilic green microalga that grows in acidic mine drainage waters contaminated by heavy metals [69]. The tolerance capacity is attributed to the capture of metals, in conjunction with the photosynthetic process and the adjustment of pH, which thus allows adaptation to high levels of metals [43]. For this reason, this strain has the potential to be used for the development of bioremediation technologies for areas contaminated with heavy metals [69].

In another study [197], by using the thermo-acidophilic microalga *Galdieria phlegrea* ACUF 784.3 in raw municipal wastewater, the removal of more than 50% of ammonium and 20% of phosphate was achieved in 24 h during the cultivation phase. It has been reported that this strain has potential for applications in bioremediation processes, due to its ability to neutralize heavy metals [197]. This result indicated that *G. phlegrea* ACUF 784.3 is a reliable candidate for water recovery municipal waste.

*Euglena gracilis* is a photosynthetic protist with the ability to resist and eliminate toxic heavy metals. In a study, this species was grown under conditions of acidic pH, anaerobiosis and with different concentrations of Cd^2+^ with the objective of determining its ability to remove Cd^2+^ and grow through the use of photosynthesis and external carbon sources. As a result, a biomass of (8.5 × 10^6^ cells mL^−1^) was obtained after 10 days of culture. This analysis determined that one of the main elimination mechanisms of this heavy metal is through biosorption (90% total removal of Cd^2+^) [198]. Similarly, Jasso-Chávez et al. (2021) [199] found that cells cultured under microaerophilic conditions in wastewater and sediment contaminated with heavy metals were able to grow and effectively remove Cd^2+^ compared to those in aerobic conditions. When compared to *Euglena mutabilis*, a protist commonly found in extreme environments, *Euglena gracilis* exhibited lower tolerance to metal exposure [200]. This dominance is attributed to *E. mutabilis* thriving in ecosystems exposed to acid mine drainage (AMD) following the mining operations at Carnoulès. Under laboratory conditions, *E. mutabilis* demonstrated the ability to grow in the presence of 32 mM arsenite, whereas *E. gracilis* failed to grow in media containing arsenite concentrations exceeding 6.66 mM. [200]. This adaptive response of *E. mutabilis* suggests its potential use as a bioindicator for contamination by arsenic and other heavy metals [201].

A pioneering report investigated the potential of *Desmodesmus* sp. MAS1 in removing heavy metals from the environment [202]. This acid-tolerant microalga, isolated from local soil and lake water samples with high concentrations of heavy metals, was placed in a medium supplemented with heavy metals at concentrations of 0.5 to 20 mg L^−1^ for Cu and Zn, and 5 to 50 mg L^−1^ for Fe, at a pH of 3.5 for 16 days [202]. The study found that the microalga could grow in the presence of 0.5 mg L^−1^ Cu, eliminating 27% of the metal. For Fe, concentrations of 5, 10, and 20 mg L^−1^ resulted in eliminations of 79%, 82%, and 86%, respectively, while for 5 and 10 mg L^−1^ of Zn, the elimination was greater than 60%. In addition to eliminating these metals, the microalga demonstrated the ability to grow and accumulate these metals intracellularly, indicating its potential for bioremediation of heavy metal-contaminated environments.

Additionally, the halotolerant *Dunaliella bardawil* UTEX-2538 showed promising potential for bioremediation of aluminum [203]. A study conducted by Akbarzadeh and Shariati (2014) demonstrated that microalgal cells bind to the aluminum ions, causing cell agglutination and sedimentation [203]. These phenomena seem to be higher than those presented for other *Dunaliella* species, indicating its greater capability and potential of the extremophilic strain to be use for bioremediation [203]. Nevertheless, to enable the commercial application of extremophilic microalgae in heavy metal bioremediation, further research is needed to optimize their metal removal efficiency under various environmental conditions and to scale up the processes for industrial use. Additionally, developing cost-effective and sustainable cultivation systems that can be integrated into existing wastewater treatment facilities will be crucial for making this technology viable on a large scale.

#### 4.2.2. Radioactive Compounds

Radionuclide elements, which are both naturally occurring and anthropogenic, enter soil and water through various human activities. These activities include extraction and milling of nuclear fuel, nuclear weapons testing, and catastrophic nuclear events, such as the Chernobyl accident and the 2011 Fukushima nuclear power plant accident [204,205]. These significant amounts of radionuclides released into the environment pose a serious threat to both ecosystems and humans [206]. Radionuclides exposed to embryonic cells demonstrated increased levels of oxidative stress and apoptosis rate, which might negatively affect growth, yet long-term studies are required [207].

However, previous studies have shown that several species of microalgae have potential for radioactive compounds removal from the environment. For instance, in the context of cesium removal, one of the most dangerous radionuclides due to its transferability, high solubility, long half-life and easy assimilation by living organisms [208], the unicellular extremophilic microalga *Galdieria sulphuraria* 074 W has demonstrated significant efficacy. In a potassium-deficient medium containing 30 μg L^−1^ of Cesium, this red microalga recovered 52 ± 15% of the cesium within 10 days. This study demonstrates that *G. sulphuraria* 074 W is a promising candidate for decontamination of radioactive cesium, using mixotrophic conditions [174].

Another investigation highlighted the extreme resistance of the extremophile microalga *Coccomyxa actinabiotis nov*. sp to ionizing radiation, along with its highly effective ability to absorb toxic metals and radionuclides, allowing its use in environments with high levels of radioactivity [209]. Silver significantly contributes to γ-emitting radioactive contaminants found in liquid effluents and nuclear-pressurized water reactors, accounting for up to 48% of the released γ-emitting radionuclides [210]. Face this situation, *C. actinabiotis* can absorb and cope with stable and radioactive silver, which results in large amounts of silver remaining confined within the microalgae due to its surface-to-volume ratio [209,210]. Therefore, this microalga is an ideal candidate for bioremediation applications of silver-contaminated waters.

### 4.3. Bioremediation of Emerging Contaminants (ECs)

Emerging contaminants (ECs) are primarily synthetic organic chemicals characterized by bioaccumulation properties and biodegradation resistance, in addition to their effects on the reproductive systems of aquatic organisms [211]. Some of the best-known emerging contaminants are from different category sources such as pharmaceuticals (PhCs), personal care products (PCPs) and endocrine-disrupting chemicals (EDCs) [212,213]. Structural complexity and low water concentration are the underlying reasons for the challenges associated with these compounds. The chemical structure of ECs defines the ability of these contaminants to be absorbed on the surface of the microalgae. In this sense, if the ECs are cationic and hydrophobic, they are attracted to the surface of the microalgae cell through electrostatic interactions, whereas if ECs are hydrophilic are repelled [214,215].

#### Pharmaceutical’s Products (PhACs)

PhACs encompass a varied range of human and veterinary medications, comprising various active pharmaceutical compounds and additional additives [216]. Despite being commonly present in water at low levels (ng/L to μg/L), these contaminants can accumulate through biomagnification in the food chain [217,218]. Its accumulation can result in adverse effects on both wildlife and humans due to the persistent and hydrophobic properties of these substances [219]. The primary four pharmaceutical classes, out of the 24 identified in water sources include nonsteroidal anti-inflammatory drugs (NSAIDs), anticonvulsants, antibiotics, and lipid regulators [216]

Different studies have been carried out on the bioremediation of PhACs microalgae. In a study, the effectiveness of the halotolerant microalgae *Nannochloropsis* sp. was evaluated to eliminate four drugs (paracetamol, ibuprofen, olanzapine, and simvastatin), both in the form of free and immobilized cells. The results demonstrated that *Nannochloropsis* sp. in free cells was effective in the elimination of olanzapine, while immobilized cells showed efficacy in the elimination of paracetamol and ibuprofen [220]. Based on the findings of this research, it can be considered a *Nannochloropsis* sp. as a promising species for the removal of pharmaceuticals from effluents.

It should be noted that the application of bioremediation technology to eliminate pharmaceutical contaminants in natural environments has not been widely implemented. Furthermore, one of the great challenges is that these compounds are distributed in various large areas, making it difficult to address their entirety. The degradation of these contaminants using technologies such as bioremediation with microalgae could have great potential, however, more studies are needed on the development of this technology [221].

**Figure 4:**
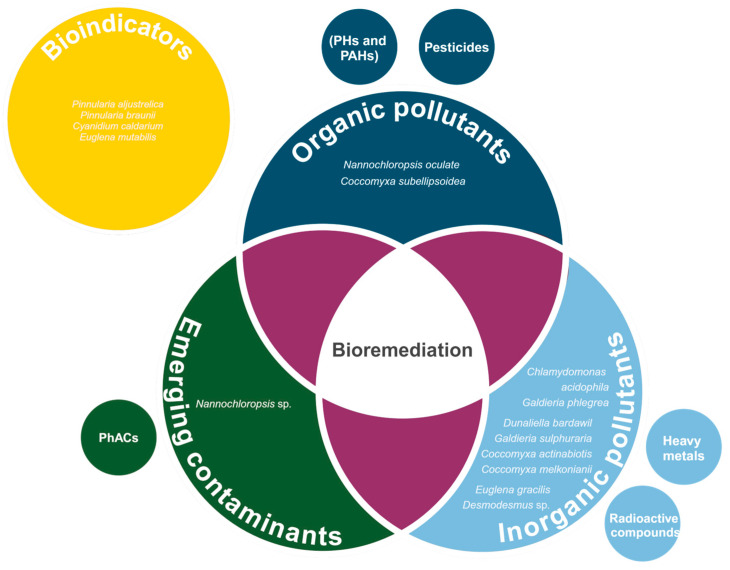
Biotechnological applications of extremophilic microalgae in bioremediation and as bioindications. Venn diagram circles represent a summarized classification of the bioremediation type. Smaller circles represent examples of pollutant molecules. Strains of species are ignored for simplification, please refer to Table 1 for respective strains and reference. Created with BioRender.com.

Overall, the implementation of extremophilic and extremotolerant microalgae species seems like a promising approach for a cost-efficient and environmental-friendly method. The vast metabolic diversity of microalgae allows these microorganisms to biodegrade and bioremediate several types of compounds (Figure 4). Microalgae efficiency for bioremediation and potential use of their end-products (biomass) posit them as promising for this biotechnological application.

## 5. Renewable Energy Sources

Energy has played a key role forming our civilization as we know it today. Until date, the main sources of energy have been non-renewable, based on fossil fuels that produce greenhouse gas emissions (GHGs) [222]. The increasing demand for energy is proportional to the rising of population, therefore, more fossil fuels are consumed over the years. This led to the ongoing global climate crisis. As an alternative to non-renewable sources, bio-energies such as bioethanol, biohydrogen, biogas and biodiesel based on microalgae feedstocks have emerged [223]. All these biofuels are also called “green energy”, part of a new “green technology”.

Biofuels can be divided in four generations: (1st) those obtained from conventional crops (e.g., sugarcane, corn, vegetable oils), (2nd) produced by by-products or waste-products of the conventional crops (e.g., used vegetable oil, solid waste, agricultural waste), (3rd) based on microbes as feedstocks (e.g., microalgae), and (4th) obtained from genetically modified microorganisms [223]. Although first- and second-generation biofuels have work as pioneer renewable energies, their main limitation relays on the insufficient supply of feedstock to meet the energy demand, and competition for agricultural land [224,225]. Microalgae biomass, a third-generation biofuel feedstock, have fast growth rates and a higher yield per space than conventional crops [226]. Additionally, they can be cultivated all over the year, in all types of weather, even on non-arable land, dismissing the competition against agricultural crops [227].

Microalgae can store high quantities of desirable macromolecules for biofuels production (polar lipids and carbohydrates). The cellular composition of the overall microalgae consist of 20–40% of lipids, 30–50% of proteins, 0–20% of carbohydrates and 0–5% of nucleic acids [228]. Although, these percentages vary significantly depending on the species. Especially, polar lipids are those naturally produced for cell growth, such as unsaturated fatty acids that play a key role in biofuel production [229]. It is thought that lipid production on microalgae is at least 6.5 times greater than common crops, stablishing them as promising feedstock sources [230].

Biochemical composition is not only dependent on the species, but also on the environment the microalgae grow naturally and is cultured later in laboratory [231]. Extreme environmental conditions have demonstrated to change and increased the biochemical composition of microalgae [232]. For example, a mesophilic strain of *Isochrysis galbana* showed an increased lipid percentage while growing at 40 ppt salinity (15.68% dry weight, DW), in comparison to 20 ppt salinity (2.80% DW) [233]. This indicates that exposure to stressors is effective in producing high-value compounds. In this sense, extremophilic and extremotolerant microorganisms, adapted to these hazardous conditions, showed a comparable productivity performance with neutrophilic species for biomass production [234]. Some studies revealed highly promising extremophilic *Dunaliella* and *Galdieria* species as biofuel feedstock [235,236]. Some of these species will be addressed throughout this section.

The main limitations of third-generation biofuels are related to how expensive is to maintain a microbial culture without undesirable contamination, and the overall cost of the traditional downstream processing [237,238]. However, the growth conditions of extremophilic and extremotolerant microalgae naturally prevents contamination [32]. For example, *G. sulphuraria* ACUF 64, an acidophilic microalga isolated from Ciavolotta sulfuric mine in Italy, showed optimal growth rates and biomass productivity under 1.7 pH conditions [236]. As most microorganisms are unable to survive under such acidic conditions, *G. sulphuraria* ACUF 64 is a promising microalga for biofuel feedstock in outdoor cultivation ponds.

Additionally, the application of biological alternatives for pretreatments has helped to lower the costs, energy required and by-product pollution of the downstream processing [239]. Pretreatments are a key part of biofuel production; they disrupt cell walls and allow the extraction of the interest compounds. Biological pretreatments are based on the usage of microorganisms or hydrolytic enzymes from bacteria with algicidal properties to facilitate disruption [239]. Additionally, the implementation of nanoparticles has gotten the scientific community’s attention. Studies found an increasing biomass, lipid content, conversion capacity into biofuels when nanoparticles are used, stabilizing the cost-effectiveness relation [240].

These advantages establish microalgae as a sustainable feedstock to meet the demand and lower the environmental impact of the energy industry. Examples of specific species of extremophilic microalgae are present for biodiesel, biogas, bioethanol and biohydrogen production in the literature. A detailed description of the extremophilic microalgae species with promising application for each biofuel synthesis is found in the subsections below.

### 5.1. Biodiesel

Biodiesel is obtained from the lipids, specifically fatty acid methyl esters (FAMEs). Previously, the crops used to extract FAMEs were exploited and required much land to grow, being associated with food crisis in developing countries [241]. Microalgae FAMEs are abundant and are extracted as crude oil and later transformed into biodiesel through transesterification by alcohols, acid catalyst, alkali catalyst, biocatalyst (through lipases) or nano-catalyst (using nanoparticles) [242,243]. This ex-situ process is simple, although the cell disruption for oil extraction is demanding. In order to facilitate biodiesel production, an in-situ methodology was developed. This consists in a “one-pot” method that disrupts the cells and performs the transesterification process simultaneously, resulting in a higher FAMEs yield [244]. This later technique increases the economic viability of microalgae-based biodiesel production. Additionally, actual car engines are compatible with biodiesel, avoiding the need for a modification, favoring the feasibility and potential application of this biofuel [245].

Several extremophilic and extremotolerant species have been described as promising feedstock for biodiesel and biogas production. This potential is possibly attributed to the lipid profile of the cell changing as an adaptation response to hazardous conditions [246]. Although lipid composition might be modified in a wide range of forms, some microalgae are known to increase the total lipid cell content. For example, *Acutodesmus obliquus* MR is a psychrotolerant microalga able to rise the lipid percentage up to 42.3% when cultured under 5% CO_2_ concentration at 10 °C [247]. Also, its lipidic composition demonstrated a higher amount of unsaturated lipids, which increases the biodiesel quality and the biotechnological potential of the microalga [247].

Moreover, *Dunaliella tertiolecta* CCAP 19/30, an alkali- and halophilic strain, evidenced a fast growth rate and high final biomass with remarkable lipid cell content [235]. These biochemical characteristics were obtained when the microalga was cultivated with a mixture of NaHCO_3_ + Na_2_CO_3_ (30 and 10 g L^−1^, respectively) [235]. Other halophilic microalgae from the *Dunaliella* genus have also been studied. *D. viridis* Teod. showed an accelerated growth rate and high lipid cell content in a mixotrophic medium supplemented with 5 mM of sodium nitrate and 5.0 g L^−1^ of glucose [248]. Both species are considered promising for biofuel production, especially biodiesel, as the high sodium concentration would inhibit biogas methanogenesis [249].

Some microalgae species from extreme environments have been studied for a more general application in both biodiesel and biogas industries. *Pseudochlorella* sp. YKT1 is an acidophilic strain with a fast exponential growth rate and high lipid content in the stationary phase (~30%) [250]. The microalgae productivity is comparable to neutrophilic commercial strains, with the advantage of growing at pH values of 3.0 to 5.0, minimizing the chance of contamination on large-scale production [250]. Additionally, a *Chlorella sorokiniana* strain with thermophilic characteristics and tolerant to high CO_2_ concentrations has been explored. When *C. sorokiniana* was cultured with 10% CO_2_ concentration, a high final biomass volume and lipid content (44.9%) was obtained [251]. The lipid content and biomass productivity of *Pseudochlorella* sp. YKT1 and *C. sorokiniana* promote the idea of both species as promising feedstocks for biodiesel and biogas production.

### 5.2. Biogas (“Biomethane”)

Biogas is the product of anaerobic digestion (AD) of a mixture rich in organic compounds such as lipids, carried out by methanogenic bacteria. It is composed mainly by methane and carbon dioxide, but H_2_, H_2_S, N_2_ and water vapor have been commonly detected and considered as impurities [252]. The biogas production process involves: (i) hydrolysis, (ii) acidogenesis, (iii) acetogenesis, and (iv) methanogenesis; in which, the hydrolysis of the cell wall is the most challenging step [253]. Prokaryotic microalgae (cyanobacteria) have a less rigid cell wall; therefore, they are preferred over eukaryotic species [254]. Additionally, and as mentioned, methanogenesis can be inhibited by sodium, making freshwater species more promising feedstocks [249].

The microalgae *Pseudochlorella* sp. YKT1 and *C. sorokiniana* are potential species for biogas production as well. Due to their high lipid content and fast rate growth, leading to a high biomass productivity [250,251]. However, some species of microalgae have shown specific advantages for biogas production. A study evaluating different extremotolerant microalgae isolates identified promising prospects [255]. *Desertifilum tharense* and *Phormidium animale*, both thermo- and alkalitolerant, presented faster growth rates and higher dry weight than microalgae isolated from neutrophilic environments [255]. During a biochemical methane potential analysis, *D. tharense* and *P. animale* evidenced the highest values (308 and 293 mL CH_4_ g VS^−1^, respectively) [255]. This biochemical test state and support the idea of both species as potential feedstocks for the biogas production, although, the authors considered *D. tharense* as the most suitable specie due of its higher biomass [255].

### 5.3. Bioethanol

Bioethanol is based on an alcoholic fermentation process, which requires microalgae with high percentages of carbohydrates, rather than lipids. The microalgae’s sugars are fermented by bacteria such as *Zymamonas mobilis* or yeast, *Saccharomyces cerevisiae* [256]. The biomass processing is rigorous as the other microbes cannot degrade the complex sugars of the microalgae [257]. Therefore, heat, alkaline, acidic, and enzymatic pretreatments are common to break down complex sugar into monomers, such as glucose [257]. Most of these processes are done separately from the fermentation, increasing the costs. As an alternative, recombinant species of yeast coding for cellulases and amylases have been used for alcoholic fermentation, showing promising results [258,259].

Microalgae from extreme environments are able to modify their sugar compositions, conferring on them potential for the production of other types of biofuels, such as bioethanol and biohydrogen. A screening study identified a thermophilic microalga tolerant to high CO_2_ concentration with a high biomass when cultured with 5% CO_2_ [251]. The microalga was annotated as *Asterarcys quadricellulare* and its biochemical analysis showed a 71.4% of total carbohydrate contents at the stationary phase [251]. This high percentage of sugars in its biochemical composition promotes the idea that *A. quadricellulare* might be a suitable microorganism for bioethanol and biohydrogen production. Moreover, *Picochlorus renovo*, a halophilic and thermotolerant species, has a fast-doubling time possibly due to cell division occurring during light and dark periods [260]. *P. renovo* presented a biomass productivity higher than the relevant large-scale production species *Nannochloropsis oceani* KA32 and *N. salina* CCMP 1776, and the recommended for biofuel production by Davis et al. (25 g m^−2^ day^−1^). Also, biomass composition analysis determined a high percentage of hydrolyzed monomeric sugars (59.5%) [260,261]. The high productivity of the strain and high quantity of monomeric sugars proposes *P. renovo* as a potential source feedstock for bioethanol and biohydrogen production.

### 5.4. Biohydrogen

Hydrogen gas is considered the energy source of the future as it causes no pollution and have a higher energy density than other fuels [262]. Biohydrogen production is carried out by microalgae working as biocatalysts (direct or indirect bio-photolysis) or as feedstock (photo- or dark fermentation). In the direct bio-photolysis, microalgae use hydrogenases to produce H_2_ [263]. Indirect bio-photolysis is performed the process when eukaryotic microalgae fixate the CO_2_ into carbohydrate which are later used for hydrogen synthesis [263]. Moreover, prokaryotic cyanobacteria produce biohydrogen during the nitrogen fixation process in specialized cells called ‘heterocysts’ by the nitrogenase enzyme [263]. Both direct and indirect bio-photolysis have low efficiency, due to the enzymes being highly sensitive to oxygen, which inhibit the process [264].

To overcome the low efficiency, researchers presented the possibility of using microalgae as feedstock. For this, biomass is fermented by bacteria producing organic acids (dark fermentation), and those organic acids are later transformed into H_2_ and CO_2_ by photosynthetic bacteria under anaerobic conditions (photo-fermentation) [263]. Remarkably, the production of biohydrogen is more thermodynamically favored under thermophilic (45–60 °C) and strict anaerobic conditions, therefore, requiring the utilization of extremophilic species [265].

In this sense, some species of extremophilic and extremotolerant microalgae are considered promising for biohydrogen production. As mentioned in the subsection above, the thermophilic and thermotolerant species *Asterarcys quadricellulare* and *Picochlorus renovo* are among these species. Due to their high productivity and carbohydrate percentage, both microalgae are here proposed as potential feedstock for biohydrogen production [251,260]. Other extreme microalgae have also demonstrated potential biotechnological applications in the biohydrogen industry. For example, *Galdieria sulphuraria* CCMEE 5587.1 is an acidophilic strain characterized by a low lipid content that renders it unsuitable for biodiesel or biogas production [266]. However, its high heating value is comparable to highly productive *Chlorella vulgaris* strains and other lignocellulosic biomass [266]. This suggested the potential of the CCMEE 5587.1 strain as feedstock for biohydrogen production by pyrolysis and gasification, processes converting organic matter (in this case the microalgae biomass) into gasses with or without combustion, respectively [266]. This hypothesis was validated by essays showing a high hydrogen production at 600 °C and 500 °C during pyrolysis and gasification trials, respectively [266].

**Figure 5:**
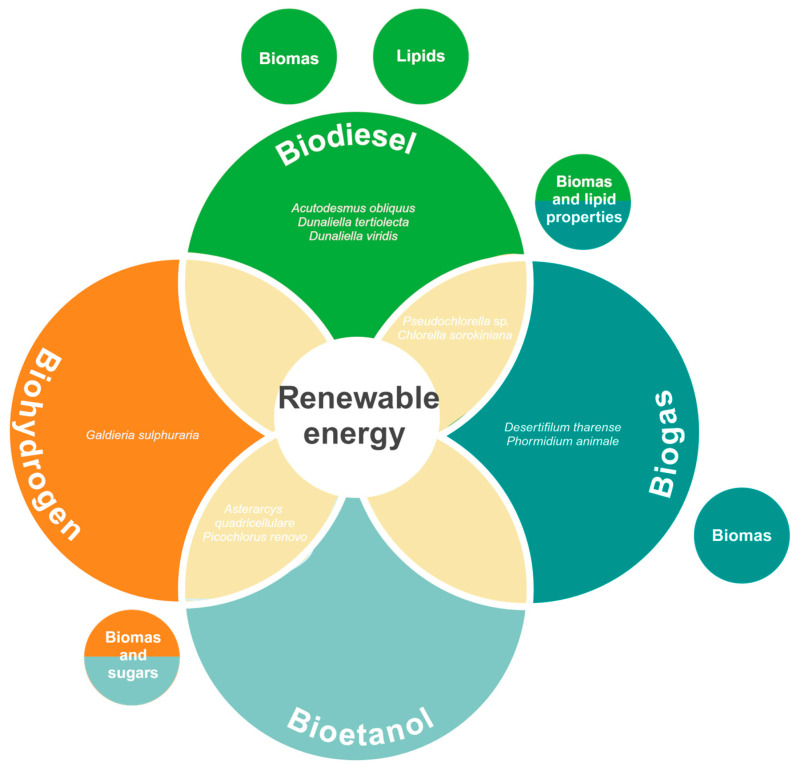
Biotechnological applications of extremophilic microalgae in the textiles and cosmetic industries. Venn diagram circles represent a summarized classification biofuel type. Smaller circles represent examples of the feedstock used for production. Strains of species are ignored for simplification, please refer to Table 1 for respective strains and reference. Created with BioRender.com.

Overall, microalgae species isolated from extreme environments are promising prospects for the biofuels industry (Figure 5). Their application as an alternative to non-renewable energy sources helps overcome several challenges related with conventional biofuels sources. Microalgae-based biofuels evade the fight against agricultural crops, meets the energy demand and reduces the greenhouse gas emissions, therefore, battle the global climate crisis. Here we described those few species found in actual literature, although more undiscovered potential strains might be ubiquitous in the environments. Hence, we encourage researchers to explore extreme environments in the search for promising microalgae species with biotechnological application in the energy industry.

## 6. Pharmaceutic and Therapeutic Industries

Pharmaceuticals are one of the most research-intensive industries in our society. Scientific progress generates a continuous flow of new products that save lives and improve and the quality of it [267]. High value pharmaceuticals and their industrial marketing can be considered the key to a multi-billion-dollar industry [268]. However, in recent years, the methods, techniques, and protocols for screening active compounds for the manufacture of new drugs have been redirected. Bioprospecting has been the key to the expansion of the drug business. Many bioactive components of organisms have been studied and identified for clinical characterization, production, and commercialization [269]. For example, cryptophycin, a cytotoxic molecule from *Nostoc* sp. GSV224, has entered preclinical and clinical trials for cancer treatment, hence being considered one of the most promising natural products from microalgae [270].

Extremophilic microalgae have aroused great interest in this field. This new attraction is driven by a mounting number of studies demonstrating the benefits that microalgae can bring to human health. Products derived from microalgae can solve or treat many of today’s clinical problems [271]. These algae are known to produce high value bioactive compounds with great benefits for human health. Microalgae are highly resistant organisms capable of inducing various stress defense mechanisms to obtain survival advantages through the production of secondary metabolites, with their own characteristics and properties [272]. Some of these metabolites and their use in the pharmaceutical industry will be highlighted throughout the section.

Carotenoids, polyunsaturated fatty acids, phenolic compounds, terpenes, and sulphated polysaccharides are examples of secondary metabolites that have been associated with antimicrobial, anti-inflammatory, aggregative, vasoconstrictor, antitumor, hypocholesterolaemia, antioxidant, immunosuppressive, antiviral properties, among many others [273,274,275]. Therefore, this section discusses the applications of different microalgae in the most important areas of research in humans, like ocular health, anti-cancer properties, and antimicrobial and antioxidant activity.

### 6.1. Ocular Health

The retina of our eyes transforms light into neural signals, which are then processed by the brain. Before light is converted, it passes through the inner retinal layers containing the oxygenated carotenoids lutein and zeaxanthin (macular pigments). The presence of these pigments gives the central retina or macula a yellow appearance, hence its clinical name macula lutea [276]. These macular pigments have been shown to influence visual performance through certain optical mechanisms, such as optical filters). Lutein and zeaxanthin absorb forward scattered shortwave light, thereby reducing glare accommodation in the eyes. They also reduce photo-stress recovery time and improve contrast [277]. In general, these pigments can prevent and reduce retinal degeneration in our eyes.

Humans do not synthesize carotenoids [276]. The main source of lutein and zeaxanthin in our body comes from the food we consume in our daily diet. For example, pea, lettuce, green pepper, broccoli, carrot, and red pepper are commonly consumed ailments that are high sources of lutein [278]. Therefore, an adequate intake of these macular pigments influences the visual performance of people through different optical mechanisms [33]. There are a variety of sources for marketed lutein. Flowering plant, such as marigold (*Tagetes* sp.), are one of the main sources of currently commercialized lutein [279]. However, flower harvesting is seasonal, and the extraction process is labor intensive [279]. Studies have shown that microalgae generate lutein production and its downstream processing require less time and consumes fewer resources, generating more pigment in a more cost-efficient manner [280].

The extremophilic alga *Chlamydomonas acidophila* RT46 is reported to be one of the microalgae with the highest cumulative lutein concentrations, with about 10 g/kg dry weight, produced in batch systems. This green algae strain is acidophilic and was isolated from Tinto River water. The river flows through a mining area of Huelva in Spain. The reported pH of this river oscillates between 1.7 and 3.1 values throughout the year [93]. The use of this strain for lutein extraction has much potential and path to be studied. Not without adding that microalgae such as *Chlamydomonas* sp. JSC4 and *Dunaliella* sp. ST10 have also been reported to be rich in macular pigments (lutein), which may represent an asset for the business in the industry [281,282].

### 6.2. Anti-Cancer Activity

Several studies have shown that there is an anti-cancer activity related to the presence of certain carotenoids, preventing different types of human cancer, like bladder, breast, hepatic, intestinal, leukemic, lung, oral, and prostate cancer [131,283]. Studies have shown that canthaxanthin has these powerful properties that could help prevent one of mankind’s most problematic diseases. The canthaxanthin treatments consist of inducing apoptosis in human colon adenocarcinoma cell lines (WiDr cell line) and human melanoma (SK-MEL-2 cell line) [284]. A dose of 10mM canthaxanthin for 48 h generated apoptosis in 18% of WiDr cells and 20% of Sk-MEL-2 cells [285].

Colon adenocarcinoma is a major form of colorectal cancer. It is the most frequent malignant pathology of the gastrointestinal tract, causing more than half million deaths in a year worldwide [286]. On the other side, human melanoma is a type of cancer that originates in the skin and usually begins with a mole following a mutation in cellular DNA related to UV exposure [287]. As is known, microalgae are a source of these anticancer carotenoids (such as canthaxanthin). The strain *Dactylococcus dissociatus* MT1, is a microalga that can survive in conditions of intense solar radiation and seasonal temperature variations. It was isolated from the Sahara Desert in Algeria and under stress conditions the algae can increase the production of canthaxanthin, which leaves open the possibility of using these microalgae for anticancer drugs in future studies [106]. Another path that can be explored is the use of *Chlorella zofingiensis*, as it is an alga recognized for its production of ketocarotenoids, such as canthaxanthin [288].

Phytosterols are other compounds with bioactivity against human tumors. As an example, ergosterol peroxide has been shown to have an inhibitory effect on the cell growth of human MCF-7 mammary adenocarcinoma cells [289]. In related studies on this steroid with extremophilic microalgae, it has been reported that with 2 micromoles of ergosterol peroxide extracted from *Chlorella vulgaris* KNUA007, there is a 77% reduction of tumor progression by tissue polypeptide antigen (tumor marker for bladder cancer) and DMBA (chemical that increase breast cancer) in mice [290]. Besides *C. vulgaris* KNUA007, there are other strains of extremophilic microalgae that have a possible potential for anticancer activity, thanks to their phytosterol content and production. *Dunaliella salina* and *Dunaliella tertiolecta* are two halophilic algae, with good performance in sterol production at specific concentrations of salt in the environment [89].

### 6.3. Antimicrobial and Antioxidant Activity

Astaxanthin is the most potent antioxidant discovered to date. This molecule acts against oxidative damage by interrupting the chain reactions of free radicals or reacting with them to produce harmless products. Other properties attributed to astaxanthin are its anti-inflammatory, anti-diabetic, gastroprotective, hepatoprotective and cardioprotective effects [291]. This valuable carotenoid is already marketed as a nutritional and health supplement or as an ingredient for feed production and has been suggested as one of the keys and most promising food ingredients of the future [100].

Normal physiological function requires a balance between free radicals and antioxidants. Antioxidants can be produced by the human body in situ, but most of them, such as carotenoids, are incorporated through diet [292]. *Haematococcus pluvialis* is the known organism that naturally produces the highest concentrations of astaxanthin as it can accumulate up to 5% of astaxanthin on a dry weight basis. The accumulation of astaxanthin in cells of *H. pluvialis* when subjected to stress conditions suggests that other microalgal cells that naturally grow under stress conditions could also produce and accumulate this valuable carotenoid [293]. Other microalgae recognized for its production of astaxanthin is *Chloromonas polyptera*, which was isolated from Antarctic snow [294].

Also, *H. pluvialis* has been shown to be related to the antimicrobial (antibacterial) activity of several microorganisms such as *Staphylococcus aureus*, *Aspergillus niger* and *Escherichia coli*. According to a study, the efficiency of this strain against these bacteria obtained better results in the red phase and using ethanol as extraction solvent. In order to identify the compound of interest, a GC-MS characterization of all the extracts obtained was performed. It was determined that this effect is related to the presence of short chain fatty acids [295]. Most microorganisms are sensitive to organic acids [296]. Short chain fatty acids have the ability to diffuse through the cell membrane, modifying the intracellular pH and the metabolism in the bacterial cytoplasm [297].

Along the same vein, promising results in antibacterial activity have been obtained with the thermophilic microalgae *Nostoc linckia*. Studies reveal their antibacterial potential against species such as *Staphylococcus aureus* and *Streptococcus mutans*, which is possibly related to their high phenol content. Just like short-chain fatty acids, these organic compounds are capable of damaging the bacterial membrane, inhibiting virulence factors and suppressing the biofilm formation [298,299]. Highlight the importance of targeting pathogens such as *S. aureus*, due to its impact on human health. These organisms significantly increase the risk of nosocomial infection, especially in hospitalized and immunocompromised patients, which represents a risk especially in the spread of drug-resistant strains [300].

Likewise, *Tetraselmis* sp. KCTC 12236 BP, a halotolerant strain, has also proved effective in its antimicrobial activity. In this case, their bioactivity is associated with antifungal properties, as it inhibits the growth of *Candida albicans* species, a human pathogen that can cause life-threatening systemic infections [301,302,303]. The water-soluble polysaccharides extracted from *Tetraselmis* sp. are responsible for this performance, due to their antioxidant action [303]. On the other hand, related to antiviral properties, studies have shown that the tannins and sulfated polysaccharides of *Arthrospira platensis* (formerly, *Spirulina platensis*) are possibly responsible for antiviral activity. This is a halotolerant strain isolated from River Krishna in India [304,305]. Its action was demonstrated with Herpes simplex-virus-type 1 (HSV-1, DNA virus), where the best results were obtained, and with Hepatitis-A-virus-type-MBB (HAV-MBB strain, RNA virus) [304].

**Figure 6:**
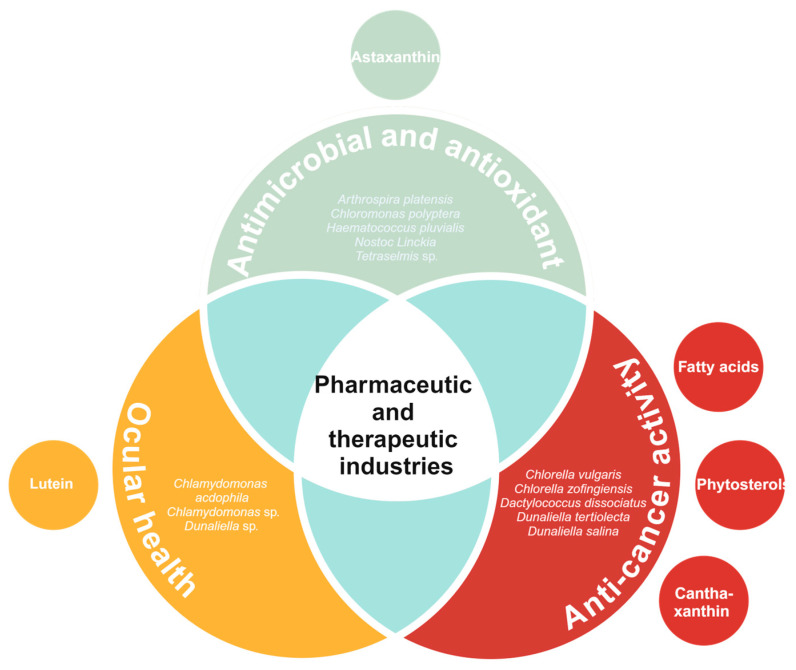
Biotechnological applications of extremophilic microalgae in the pharmaceutical industry. Venn diagram circles represent a summarized application. Smaller circles represent examples of molecules responsible for bioactive potential. Strains of species are ignored for simplification, please refer to Table 1 for respective strains and reference. Created with BioRender.com.

In summary, microalgae species, as the ones here discussed, are able to synthetize a vast diversity of primary and secondary metabolites. Some of these compounds (e.g., fatty acids, carotenoids) have reported bioactive properties with potential uses in the pharmaceutical industry (Figure 6). Adapting to extreme environments might be one of the factors involve in the biosynthetic potential of these extremophilic and extremotolerant microalgae, a phenomenon previously demonstrated in bacteria [306].

## 7. Future Directions, Challenges, and Conclusions

The use of microalgae biomass by the food industry faces several challenges, including the need to comply with stringent government regulations about food safety, high production costs, scalability of processes, and consumer acceptance of microalgae as a viable food source [35,61]. Overcoming these hurdles requires significant research and development efforts. One of the biggest challenges is the development of efficient and profitable systems for the production and recovery of energy from microalgae, which necessitates increasing productivity and improving efficiency in the recovery of the generated biomass [44]. To achieve this, it is imperative to promote the development and optimization of these systems, aiming to enhance both economic efficiency and sustainability in the use of microalgae [307].

Future directions in this field should focus on improving cultivation techniques, such as optimizing light, nutrient supply, and CO_2_ concentration to maximize microalgae growth and biomass yield. Advances in genetic engineering could also play a crucial role in developing strains with higher productivity and better nutritional profiles. Additionally, integrating biorefinery concepts to utilize every part of the biomass can improve economic viability by producing multiple high-value products from a single source. Moreover, increasing public awareness and acceptance of microalgae-based products is essential. This can be achieved through education about the environmental benefits and nutritional value of microalgae, alongside efforts to develop appealing and palatable food products. Collaborations between researchers, industry stakeholders, and policymakers can facilitate the creation of supportive frameworks for microalgae production and market entry.

Addressing these challenges and exploring these future directions will not only enhance the economic and environmental sustainability of microalgae production but also pave the way for innovative applications in food, pharmaceuticals, cosmetics, and biofuels. The vast variety of applications of the extremophilic and extremotolerant microalgae here explored are summarized in Table 1. The versatility of microalgae applications can lead to the creation of more environmentally friendly products compared to conventional alternatives, thereby contributing to global sustainability goals. By continuing to invest in research and development, we can unlock the full potential of microalgae as a resource for a sustainable future.

**Table 1:** Microalgae species with biotechnological applications, extremophile characteristics and place of isolation.

Biotechnological Industry	Microalgae Specie	Isolation Place	Extreme Characteristics	Biotechnological Application
Food industry	*Arthrospira platensis*	Alkaline and hypersaline lakes [58]	Alkali- and halophilic [58]	High protein content for food supplements [58]
	*Arthrospira maxima* (LIMS-PS-1691)	Alkaline lakes [121]	Alkaliphilic [122]	Biomass and nutritional compounds production [122]
	*Chlamydomonas malina* RCC2488	Beaufort Sea of Artic Ocean [74]	Psychrophilic [74]	Source of lipids and poly-unsaturated fatty acids (PUFAs) [74]
	*Chlamydomonas nivalis*	Liquid water in snow and glaciers of alpine and polar regions [101]	Psychrophilic [101]	Source of astaxanthin, β-carotene, tocopherol and lutein [101,140]
	*Chlorella vulgaris* CA1	Dairy wastewater [59]	Ammonia-tolerant [59]	High protein content [59]
	*Chlorella zofingiensis*	Fresh water [104]	Halotolerant [104]	Source of astaxanthin and lipids for nutritional supplements [103]
	*Coccomyxa acidophila*	Acidic waters of Tinto River, Spain [92]	Acidophilic [92]	Source of lutein [92]
	*Coccomyxa melkoniani* SCCA 048	Polluted mine waters of Rio Irvi River, Italy [70]	Resistant to heavy metal contamination [70]	Source of lipids with high nutritional value [70]
	*Coccomyxa onubensis*	Acidic waters of Tinto River, Spain [95]	Acidophilic [95]	Accumulation of lutein for food supplements [95,308]
	*Cyanidioschyzon merolae* 10D	Phelgrean fields, Italy [118]	Acido-, halo- and thermophilic [118,153]	Source of thermostable phycocyanin [118,153]
	*Cyanidium caldariumm*	Acid thermal area of Yellowstone National Park [117]	Acidophilic [117]	Source of phycocyanin [117]
	*Dactylococcus dissociatus* MT1	Sahara Dessert of Algeria [106]	Resistant to solar ration and extreme daily and seasonal temperature variations [106]	Source of lutein and β-carotene, and lipids with antioxidant properties [106,309]
	*Dunaliella salina*	Shambar Salt Lakes, India [310]	Halophilic [310]	Source of β-carotene [310]
	*Dunaliella tertiolecta* DCCBC26	Salt Lake of Urmia, Iran [90]	Halophilic [90]	Production of antioxidants and lipids [89]
	*Galdieria phlegrea* ACUF 009	Cryptoendolithic environments of the Phlegrean Fields, Italy [120]	Acido- and thermophilic [120]	Source of thermostable C-phycocyanin for food colorant and preservative [119]
	*Galdieria sulphuraria* CCMEE 5587.1	Unknown, obtained from Culture Collection of Microorganisms from Extreme Environments (Pacific Northwest National Laboratory, Richland, USA) [62]	Acidophilic and thermotolerant [62]	High protein content for food supplements [62]
	*Graesiella* sp.	“AinEchfa” hot spring, Tunisia [75]	Thermophilic [75]	Source of lipids with high nutrional value [75]
	*Mesataenium berggrenii*	Tiefenbach Glacier, Austrian Alps [96]	Psychrophilic [96]	Source of lutein and β-carotene [96]
	*Microchloropsis gaditana* CCMP526	Gippsland Lakes, Australia [65]	Halotolerant [65]	Protein fortification in food [64]
	*Synechococcus* sp. PCC 6715	Hot springs Yellowstone, USA [115]	Thermophilic [115]	Source of thermostable phycocyanin [115]
	*Synechococcus vulcanus*	Hot springs in Yellowstone National Park, USA [311]	Thermophilic [123]	Source of phycocyanin [123]
Textile and cosmetics industries	*Aphanothece halophytica*	Isolated from Solar Lake, Sinai [146]	Halotolerant [146]	Source of mycosporine-2-glycine [146]
	*Chlamydomonas nivalis*	Liquid water in snow and glaciers of alpine and polar regions [101]	Psychrophilic [101]	Source of astaxanthin, β-carotene, tocopherol and lutein [101,140]
	*Chlorella vulgaris* BUACC25	Sonapur Sea Beach, Ganjam, Odisha [145]	Halotolerant [145]	Source of antioxidants (phenols and flavonoids) [145]
	*Chlorella vulgaris* M-207A7	Beverage Technology Research Laboratory’s culture collection [144]	Halotolerant [144]	High chlorophyll content due to induced mutation [144]
	*Chroococcidiopsis* sp. B13	Solar panels [136]	Resistant to desication, ionizing radiation and UV light [136]	Source of antioxidants [137]
	*Cyanidioschyzon merolae* 10D	Phelgrean fields, Italy [118]	Acido-, halo- and thermophilic [118,153]	Source of thermostable phycocyanin [118,153]
	*Cyanidium caldariumm*	Acid thermal area of Yellowstone National Park [117]	Acidophilic [117]	Source of phycocyanin [117]
	*Coccomyxa melkonianni* SCCA048	Polluted mine waters of Rio Irvi River, Italy [70]	Resistant to heavy metal contamination [70]	Source of lutein and high lipid content [139]
	*Dunaliella salina*	Shambar Salt Lakes, India [310]	Halotolerant [310]	Source of yellow pigment β-carotene [310]
	*Dunaliella tertiolecta* DCCBC26	Salt Lake of Urmia, Iran [90]	Halophilic [90]	Source of antioxidants and lipids [89]
	*Galdieria phlegrea* ACUF 009	Cryptoendolithic environments of the Phlegrean Fields, Italy [120]	Acido- and thermophilic [120]	Source of thermostable C-phycocyanin [119]
	*Mesataenium berggrenii*	Tiefenbach Glacier, Austrian Alps [96]	Psychrophilic [96]	Source of β-carotene [96]
	*Synechococcus lividus*	Thermal alkaline hot springs of Yellowstone National Park [151]	Thermophile [151]	Fount of C-phycocyanin [151]
Bioremediation	*Chlamydomonas acidophila* RT46	Acidic waters of Tinto River, Spain [196]	Acidophilic and resistant to heavy metal contamination [196]	Removal of cadmiun [196]
	*Coccomyxa actinobiotis*	Storage pool of element in research nuclear reactor, France [209]	Resistant to ionizing radiations and metallotolerant [209]	Bioremediation of radioactive and silver-polluted waters [209,210]
	*Coccomyxa melkoniani* SCCA 048	Polluted mine waters of Rio Irvi River, Italy (43)	Resistant to heavy metal contamination (43)	Phycoremediation of heavy metals [69]
	*Coccomyxa subellipsoidea* C-169	Marbel Point, Antarctica [312]	Psychrotolerant [312]	Degradation of organophosphates [312]
	*Cyanidium caldarium*	Littoral zone of Lake Caviahue, Argentina [185]	Acidophilic [185]	Bioindicator of Polycyclic aromatic hydrocarbons pollution [185]
	*Desmodesmus* sp. MAS1	Local soil and lake water sample [232]	Acid-tolerant [232]	Bioremediation of acid soils [313], heavy metal removal [202]
	*Dunaliella bardawil*	Sambar Lake, India [314]	Halotolerant [314]	Bioremediation of aluminum polluted environments [203]
	*Euglena gracilis*	Acid and heavy metal polluted waters [198]	Acidophilic and metallotolerant [198]	Bioremediation by heavy metal remotion [198]
	*Euglena mutabilis*	Acid mine drainage near Reigous creek, France [201]	Acidophilic and metallotolerant [201]	Bioindicator for arsenic and other heavy metal contamination [200]
	*Galdieria phlegrea* ACUF 784.3	Geothermal volcanic soils [197]	Acido- and thermophilic [197]	Municipal wastewater treatment [197]
	*Galdieria sulphuraria* 074 W	Sulfuric and acidic hot springs from Mt. Lawu, Indonesia [315]	Acido- and thermophilic [315]	Removal of cesium (Cs) [174]
	*Nannochloropsis oculate*	Unknown, obtained from the Culture Collection of Algae at the University of Texas Austin, USA [184]	Halophilic [184]	Removal of polyhydroxyalkanoates (PHAs) [184]
	*Nannochloropsis* sp.	Unknown, obtained from Varicon Aqua Solution, UK [220]	Halotolerant [220]	Removal of pharmaceuticals compounds [220]
	*Pinnularia aljustrelica*	Acidic waters of Aljustrel mining area, Portugal [316]	Acidophilic and metallotolerant [316]	Bioindicator of acid mine drainage [178]
	*Pinnularia braunii*	Streams near agricultural used lands Manyame, Zimbabwe [179]	Acidophilic and metallotolerant [179]	Bioindicator of water quality [179]
Renewable energy industry	*Acutodesmus obliquus MR*	Freshwater samples of South Korea [317]	Psychrotolerant [247]	High lipid cell content for biodiesel production [247]
	*Asterarcys quadricellulare*	Water bodies near a JSW steel plant, India [251]	Thermophilic and high CO2 tolerance [251]	High carbohydrate cell content for bioethanol and biohydrogen production [251]
	*Chlorella sorokiniana*	Water bodies near a JSW steel plant, India [251]	Thermophilic and high CO2 tolerance [251]	High lipid cell content for biogas and biodiesel production [251]
	*Desertifilum tharense*	Thermal water of Turkey [255]	Thermo- and alkalitolerant [255]	High biochemical methane potential for biogas production [255]
	*Dunaliella terticola* CCAP 19/30	Saline marine environments [235]	Alkali- and halophilic [235]	High lipid cell content for biodiesel production [235]
	*Dunaliella viridis* Teod.	Maharlu Salt Lake, Iran [248]	Halophilic [248]	High lipid cell content for biodiesel production [248]
	*Gladieria sulphuraria* ACUF 64	Sulfuric mine Ciavolotta, Italy [236]	Acidophilic [236]	High biomass productivity for biofuel feedstock [236]
	*Gladieria sulphuraria* CCMEE 5587.1	Unknown, obtained from Culture Collection of Microorganisms from Extreme Environments (Pacific Northwest National Laboratory, Richland, U.S.A.) [266]	Acidophilic [266]	High heating value for biohydrogen production [266]
	*Phormidium animale*	Thermal water of Turkey [255]	Thermo- and alkalitolerant [255]	High biochemical methane potential for biogas production [255]
	*Picochlorum renovo*	Brackish and marine waters [260]	Halophile and thermotolerant [260]	High biomass and carbohydrates productivity for bioethanol and biohydrogen production [260]
	*Pseudochlorella* sp. *YKT1*	Sulfuric mine drainage in Nagano Prefecture, Japan [250]	Acidophilic [250]	High lipid cell content biodiesel and biogas production [250]
Pharmaceutics and therapeutics industries	*Chlamydomonas acidophila* RT46	Acidic waters of Tinto River, Spain (73)	Acidophilic and resistant to heavy metal contamination (73)	Source of lutein and β-carotene [318]
	*Dactylococcus dissociatus* MT1	Sahara Dessert of Algeria [106]	Resistant to solar ration and extreme daily and seasonal temperature variations [106]	Efficient producer of canthaxanthin [106]
	*Dunaliella salina*	Shambar Salt Lakes, India [310]	Halotolerant [310]	Source of phytosterols [89]
	*Dunaliella tertiolecta*	Salt Lake of Urmia, Iran [90]	Halophilic [90]	Source of phytosterols [89]
	*Haematococcus pluvialis*	Mountainous and valley areas of the Black Sea, Caucasus and Crimea [293]	Can support high salinity [293]	Accumulates large quantities of astaxanthin and produces short chain fatty acids with antimicrobial properties [293]
	*Chloromonas polyptera*	Snow, Antarctica [294]	Psychrophilic [294]	Abundant accumulation of astaxanthin [294]
	*Chlorella vulgaris* KNUA007	Meltwater stream, King George Island, Antarctica [319]	Cold-tolerant [319]	Rich in nutritional fatty acids (cardiovascular health) [319]
	*Chlamydomonas* sp. JSC4	Ocean of southern Taiwan [320]	Halotolerant [320]	Source of lutein [281]
	*Dunaliella* sp. ST10	Hyperaline pond in the “Saline di Tarquinia” on Tyrrhenian Coast, Central Italy [282]	Halotolerant [282]	Fount of lutein [282]
	*Chlorella zofingiensis*	Fresh water [104]	Halotolerant [104]	High astaxanthin and canthaxanthin content [104,288]
	*Nostoc linckia*	Soil on both sides of the water stream of the Helwan hot springs, Egypt [299]	Thermophilic [299]	Source of phenols with antimicrobial properties [299]
	*Tetraselmis* sp. KCTC 12236 BP	Young Heung Island, Incheon, Korea [302]	Halotolerant [302]	Accumulates polysaccharides with antimicrobial activity [303]
	*Arthrospira platensis*	River Krishna, Tungabhadra, India [305]	Halotolerant [304]	Concentration of polysaccharides with antimicrobial activity [304]

## Data Availability

Data sharing not applicable. Acknowldegment: The language and grammar of certain paragraphs in this Review Article have been checked using a large scale language model.
